# Viruses interact with hosts that span distantly related microbial domains in dense hydrothermal mats

**DOI:** 10.1038/s41564-023-01347-5

**Published:** 2023-04-06

**Authors:** Yunha Hwang, Simon Roux, Clément Coclet, Sebastian J. E. Krause, Peter R. Girguis

**Affiliations:** 1 Department of Organismic and Evolutionary Biology, Harvard University, Cambridge, MA USA; 2 DOE (Department of Energy) Joint Genome Institute, Lawrence Berkeley National Laboratory, Berkeley, CA USA; 3 Department of Earth, Planetary, and Space Sciences, University of California, Los Angeles, CA USA

**Keywords:** Metagenomics, Microbiome, Bacteriophages, Biofilms, Virus-host interactions

## Abstract

Many microbes in nature reside in dense, metabolically interdependent communities. We investigated the nature and extent of microbe-virus interactions in relation to microbial density and syntrophy by examining microbe-virus interactions in a biomass dense, deep-sea hydrothermal mat. Using metagenomic sequencing, we find numerous instances where phylogenetically distant (up to domain level) microbes encode CRISPR-based immunity against the same viruses in the mat. Evidence of viral interactions with hosts cross-cutting microbial domains is particularly striking between known syntrophic partners, for example those engaged in anaerobic methanotrophy. These patterns are corroborated by proximity-ligation-based (Hi-C) inference. Surveys of public datasets reveal additional viruses interacting with hosts across domains in diverse ecosystems known to harbour syntrophic biofilms. We propose that the entry of viral particles and/or DNA to non-primary host cells may be a common phenomenon in densely populated ecosystems, with eco-evolutionary implications for syntrophic microbes and CRISPR-mediated inter-population augmentation of resilience against viruses.

## Abstract (web summary)

Metagenomics and Hi-C proximity-ligation sequencing show that viruses in dense microbial communities can interact with multiple, distantly related microbial hosts.

## Main

Most bacteria and archaea in nature are found in aggregates or as biofilms^1^. These microbial aggregates often consist of phylogenetically distant organisms engaging in interdependent metabolisms (for example, syntrophy)^2^. However, most host-virus interactions are studied in homogeneous liquid cultures and many gaps remain in our understanding of host-virus interactions in dense, substrate-bound and heterogeneous biofilms^3^. In particular, major questions exist with regard to host range, viral life cycle, modes of dispersal and host-virus co-evolution in complex microbial communities where genetically diverse and phylogenetically distant microbes co-exist in high proximity and engage in highly nested metabolisms.

Generally, viruses are thought to infect a narrow range of hosts. Recent studies, however, have suggested that broad host range viruses may be more common in nature and may have been overlooked due to cultivation biases^4^. Thus far, there exist reports of viruses infecting multiple bacterial species^5^, orders^6^ and possibly phyla^7–9^. Additionally, viral host ranges have also been shown to be a dynamic trait^10^. Notably, a recent study^11^ reported that phage adsorption and entry into cells do not equate to a full completion of the lytic cycle, indicating that viruses may interact with a more diverse set of cells in which a complete infection cycle can be performed.

We hypothesized that broader host range viruses may be prevalent in biofilms dominated by syntrophic metabolisms due to extended contact with phylogenetically diverse microbes and limited host and viral dispersal and/or habitat range caused by extracellular polymeric substances (EPS) and spatial heterogeneity. To address this hypothesis, we characterized viral genomes and any viral interactions with bacteria or archaea (hereafter referred to as host-virus interactions) in a deep-sea hydrothermal microbial mat, these mats being ubiquitous chemoautotrophic biofilms around hydrothermal vents. These mats consist of very dense, metabolically coupled communities of bacteria and archaea^12^, and feature sharp spatial gradients and temporal variability in temperature and geochemistry^13^. We show that phylogenetically distant microbes (that is, taxa from different phyla and even domains) with putatively syntrophic metabolic capacities often encode Clustered Regularly Interspaced Short Palindromic Repeats (CRISPR)-based immunity against the same viruses in the mat. This pattern is not detected from the physically adjacent hydrothermal plume samples featuring lower biomasses of metabolically similar communities. Furthermore, these microbial genomes exhibit co-localizations with the same viral genomes on the basis of Hi-C proximity-ligation sequencing. By examining publicly available metagenomes, we also found viruses interacting with both bacterial and archaeal taxa in other ecosystems known to harbour syntrophic biofilms. We further investigated the eco-evolutionary implications of these host-virus interactions by examining auxiliary metabolic genes (AMGs) in the viral genomes, as well as identifying viral and microbial genes undergoing selection. Finally, we propose four models of viral polyvalent interactions with syntrophic hosts and discuss their implications on microbial evolution, particularly with regard to horizontal gene transfer, genetic diversification and CRISPR-mediated community-wide immunological memory.

## Results

### A syntrophic and metabolically interdependent microbial mat

**Fig. 1:**
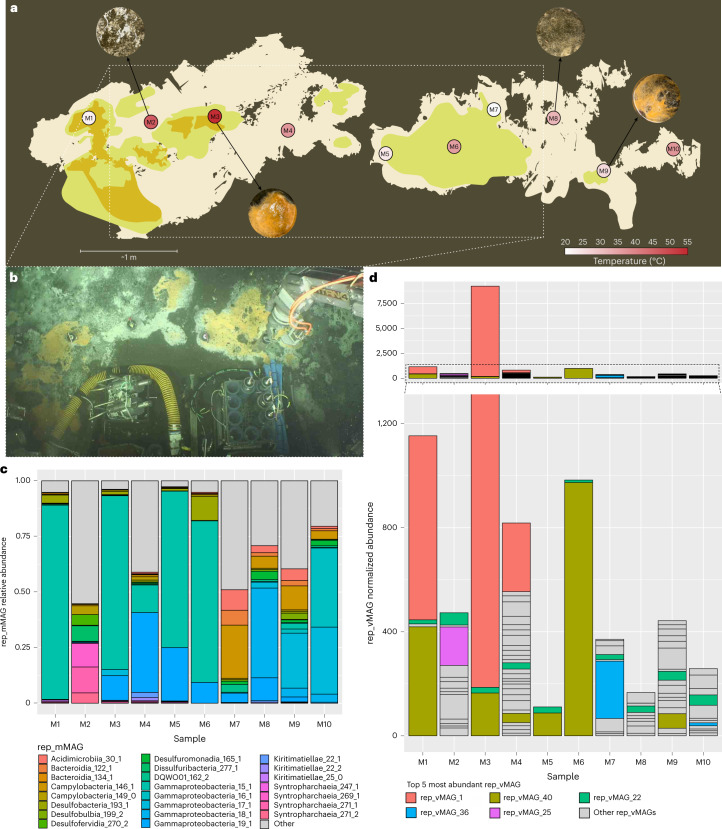
Highly heterogeneous yet contiguous deep-sea hydrothermal mat. **a**, Visual schematic of the sampled microbial mat. Sampling locations are illustrated on the basis of the three main colours (orange, yellow and white) observed during sampling. Distances and shapes are approximate and were reconstructed using the high-resolution videos and photos taken during the ROV *Jason* dive. Pushcore locations are coloured on the basis of in situ temperature. Different morphologies of some of the sampled mat materials are shown with photos taken shipboard during sampling of the pushcores. **b**, Top view of the middle section (approximately outlined as a dashed box in **a**) of the sampled mat. **c**, Relative abundances of the top 10 most abundant rep_mMAG (species-level, 97% ANI cut-off) in each sample. **d**, Normalized abundances of 47 high-quality or complete rep_vMAGs (95% ANI cut-off) in each sample. The top 5 most abundant rep_vMAGs are coloured.

**Extended Data Fig. 1:**
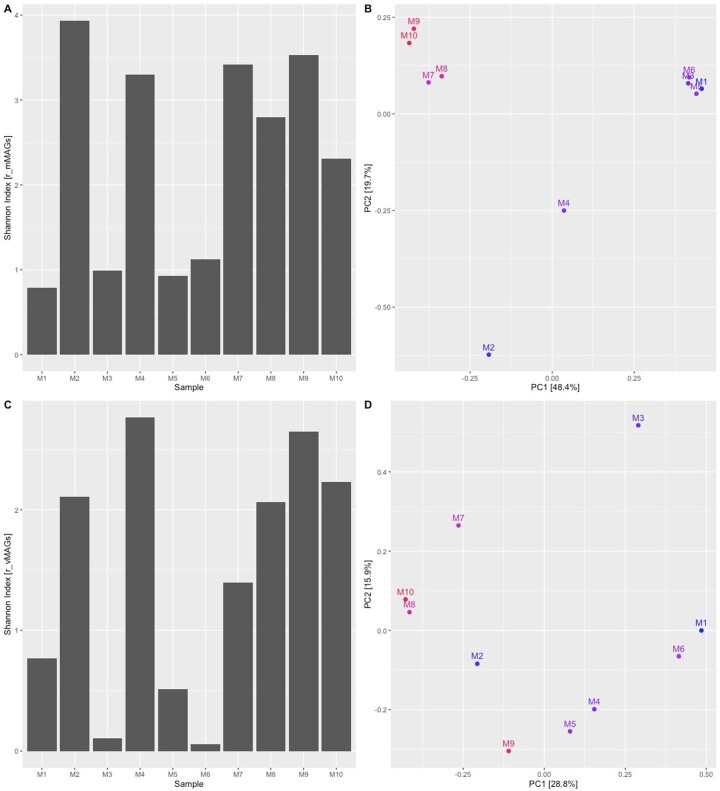
Hydrothermal mat microbial and viral taxonomic diversity and principal coordinate analysis (PCoA). Shannon’s diversity index (**A**) and PCoA (**B**) rep_mMAGs and Shannon’s diversity index (**C**) and PcoA (**D**) for rep_vMAGs in samples M1-10. PCoA plots show samples colored according to the position across the transect (leftmost: red, rightmost: blue) and the percentage of variance explained by each axis is shown in the corresponding axis labels.

Expedition RR2107 took place in the Guaymas Basin, Mexico, from 11 November to 5 December 2021. During dive J2-1398 of the remotely operated vehicle (ROV) *Jason*, 10 pushcores (7.5 cm diameter, 30 cm long) were recovered from a contiguous hydrothermal mat. The mat was heterogeneous in both temperature and chemistry, with subsurface temperatures ranging between 21 °C and 53 °C (Fig. 1a,b and Supplementary Table 1a). DNA extracted from the surficial mat and top sediment layer from each pushcore sample were used as templates for metagenomic sequencing, yielding 1.8 billion 150-bp read pairs (see sequencing statistics in Supplementary Table 1c). We recovered 303 mid- to high-quality genetically defined representative microbial metagenome-assembled genomes (rep_mMAGs) from across the mat using genome binning based on read coverage, *k*-mer frequency and/or proximity-ligation data (Methods) (Supplementary Table 2). Out of 10 samples, 8 were dominated by 5 genetically defined (97% average nucleotide identity (ANI), species-level) populations of Gammaproteobacteria, all belonging to the Beggiatoaceae family (Fig. 1c), with genomic capacities for nitrate-coupled sulfur oxidation (Supplementary Table 3). The other two samples (M2 and M7) showed higher species evenness (Shannon’s diversity index, Extended Data Fig. 1a). More than half (*n* = 153) of the populations were uniquely detected in a single sample, and only 3 rep_mMAGs (Gammaproteobacteria_19_1, Campylobacteria_146_1 and Acidimicrobiia_30_1) were detected across all 10 samples. Despite the apparent high morphological and environmental patchiness of the mat (Supplementary Table 1), the microbial community composition of the mats could be grouped into two spatially organized sets driven by the shift in the composition of abundant sulfur oxidizing bacteria (SOB) populations (Extended Data Fig. 1b), suggesting that physical proximity probably plays a bigger role in community assembly. High variability in environmental conditions (for example, temperature or hydrogen sulfide; Supplementary Table 1) may account for the sample-specific variations in rarer populations, which could be explored further with the measurements of other geochemical species (for example, methane and ammonia) that these organisms are capable of metabolizing. As previously described^14^, these microbial mats were dominated by chemoautotrophic bacteria and archaea, largely sustained by geothermally derived reduced sulfur, nitrogen and hydrocarbon compounds, with 223, 192 and 40 out of 303 rep_mMAGs encoding at least one gene involved in sulfur, nitrogen and methane metabolisms, respectively (Supplementary Table 2). Microbial metabolisms in these hydrothermal sediments are thought to be highly interdependent^15^, and previous research has found evidence of the syntrophic anaerobic methane-oxidizing (ANME) archaea and sulfate reducing bacteria (SRB)^16^ that couple anaerobic methane oxidation to sulfate reduction, as well as hypothesized sulfur-based syntrophy between SRB and SOB^17^. Notably, 76% of these rep_mMAGs encoded either a hydrogenase, c-type cytochrome and/or PilA, indicating a widespread potential for substrate-mediated and/or direct interspecies electron transfer (Supplementary Table 2).

### Characterization of near-complete viral genomes

**Extended Data Fig. 2:**
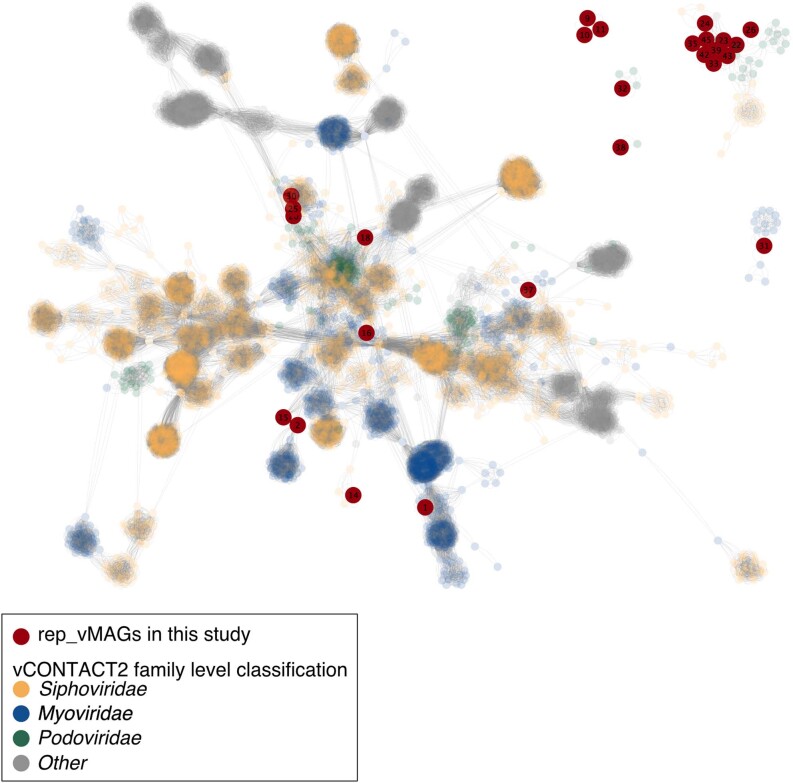
rep_vMAG taxonomic diversity relative to the reference viral genomes. Larger red nodes are viruses assembled in this study. Colors of reference viral genome nodes are according to family level classifications used in the vCONTACT2 database.

**Extended Data Fig. 3:**
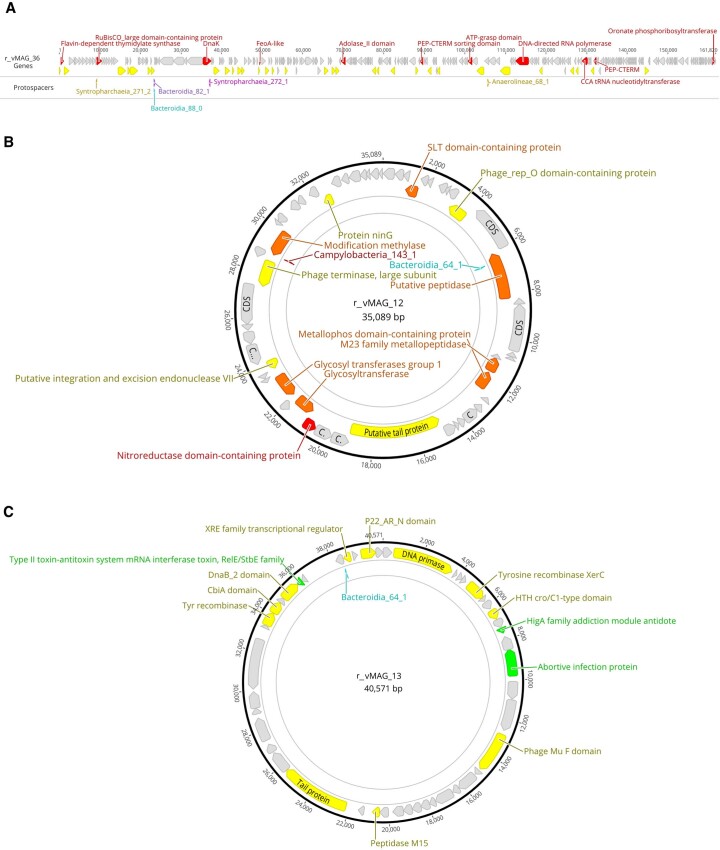
Mat viral genome annotations. Auxiliary metabolic genes and host-virus arms race in viral gene content. Putative auxiliary metabolic genes (AMGs; red) in rep_vMAG_36 (**A**) and rep_vMAG_12 (**B**), and defense system genes (green) in rep_vMAG_13 (**C**). These genes are flanked by hallmark phage genes (yellow) and other notable genes found in phages (orange). Locations of protospacers are shown in the bottom track and labeled according to the rep_mMAG the CRISPR is binned in.

Across 10 metagenomes, we recovered 47 representative viral MAGs (rep_vMAGs, dereplicated at 95% ANI) that were either complete (*n* = 27) or high-quality (*n* = 20) according to CheckV^18^ using a high-confidence completeness estimation method (see Methods for details and Supplementary Table 4 for statistics on rep_vMAGs). These rep_vMAGs varied significantly in size, ranging from 12 kbp to 437 kbp. Only two rep_vMAGs (vMAG_46 and vMAG_25) were detected as proviruses using CheckV^18^. The abundance profiles of rep_vMAGs were more heterogeneous than those of the rep_mMAGs and exhibited less proximity-based clustering (Extended Data Fig. 1d). Similar to microbial populations, more than half (*n* = 26) of the rep_vMAGs were detected in only one sample (implying a small habitat range), while only one rep_vMAG (rep_vMAG_22) could be detected across all 10 samples. One sample (M3) exhibited greater than 7-fold abundance of a lytic viral population (rep_vMAG_1) consistent with a recent viral infection (Fig. 1d). Viral diversity was highly correlated with microbial species diversity (Pearson’s correlation coefficient: 0.83, *n* = 10, *P* = 0.00293; Extended Data Fig. 1c), although no statistically significant co-correspondence^19^ (sCoCA, *n* = 10, *P* > 0.05) between microbial and viral compositions was identified. The rep_vMAGs recovered from this study exhibited very high taxonomic and gene content diversity relative to the genetic diversity space occupied by the reference viral genomes (Extended Data Fig. 2). Only 4 rep_vMAGs could be clustered at the ‘genus’ level^20^ with reference viral genomes, and could be classified as two (previously designated) *Podoviridae*, one *Myoviridae* and one *Siphoviridae* (Supplementary Table 4). Notably, a taxonomic cluster consisting of 7 rep_vMAGs was distantly associated with *Flavobacterium* phages, and 3 of the rep_vMAGs formed a novel genus-level cluster that shared no similar genes with any of the characterized reference viral sequences. A majority (29 out of 49) did not share high similarity in gene content with the reference or with each other. Many of the viral genomes contained novel auxiliary metabolic genes (AMGs) such as Rubisco large domain-containing protein, aldolase II domain-containing protein, nitroreductase domain-containing protein, phosphate starvation-inducible protein PhoH and terillium resistance protein TerD (Extended Data Fig. 3a,b). We also detected evidence of host-virus arms race, with some viral genomes encoding defence machinery such as RelE/StbE family toxin, HigA family antidote and a putative abortive infection protein (Extended Data Fig. 3c). A complete list of the annotated AMGs and other notable viral genes is provided in Supplementary Table 5.

### Microbial CRISPR-Cas loci

**Extended Data Fig. 4:**
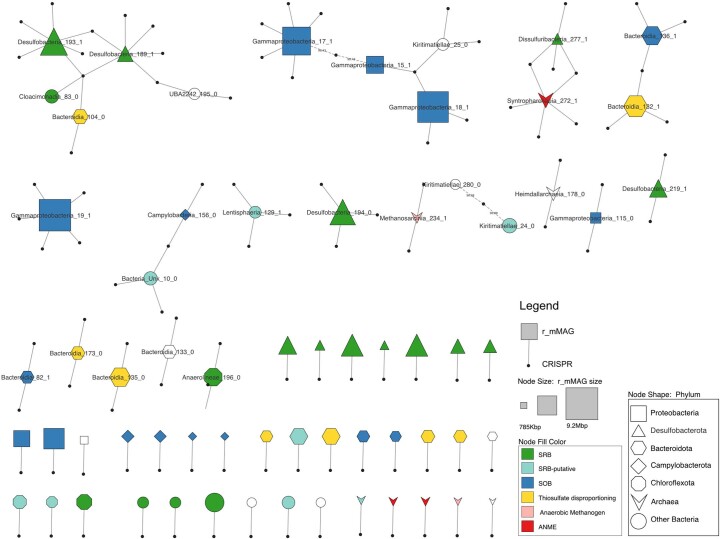
Network visualization of rep_mMAGs and their CRISPRs (repeats). Larger nodes represent rep_mMAG, where the node shape denotes the phylum the rep_mMAG belongs to, the node size is correlated to the rep_mMAG size and the node color corresponds to the genome-informed metabolic capabilities. SRB: Sulfur reducing bacteria, SOB: Sulfur oxidizing bacteria, SRB-putative: Putative sulfate reducing bacteria encoding *aprAB* and/or *dsrD*. ANME: Anaerobic methanotrophic archaea.

rep_mMAGs recovered in this study featured diverse and abundant CRISPR-Cas systems. We detected 317 *cas* loci across 119 out of 303 rep_mMAGs (Supplementary Table 6). The number of *cas* loci in a population genome varied between 1 and 16, with an ANME-1 rep_mMAG (Syntropharchaeia_272_1) encoding 16 *cas* loci belonging to diverse subtypes (6 class 1 subtype IB, 3 class 1 subtype IIIA, 2 class 1 subtype IIIC, 1 class 1 type I, 2 class 1 type III, and 2 unclassified clusters). In addition, we identified 116 unique CRISPR repeats across 65 genetically defined populations (Extended Data Fig. 4 and Supplementary Table 7). These CRISPR repeats were clustered by sequence similarity (>95% nucleotide identity (ID)) into 102 clusters. Most (91%) of the detected CRISPRs were specific to a population and 80% of the CRISPR-encoding populations were associated with at most 2 unique CRISPRs. However, we observed identical or near identical (>95% ID) CRISPR repeats shared among phylogenetically distant populations. It is possible that these CRISPR loci were horizontally transferred^21^, but we cannot rule out the possibility of binning errors resulting from their repetitive and divergent nature. Such CRISPR repeats detected across taxa were excluded from spacer-based host-virus matching due to the ambiguity in assigning a specific host taxon to a repeat. Additionally, we identified populations (Gammaproteobacteria_17_1, Desulfobacteria_193_1, Desulfobacteria_189_1) encoding as many as 6 distinct CRISPR repeats, probably representing within-population diversity of CRISPR loci. No correlation was found between the number of unique CRISPRs and the rep_mMAG size, relative abundance or habitat range.

### Reconstructing historical host-virus interactions

**Fig. 2:**
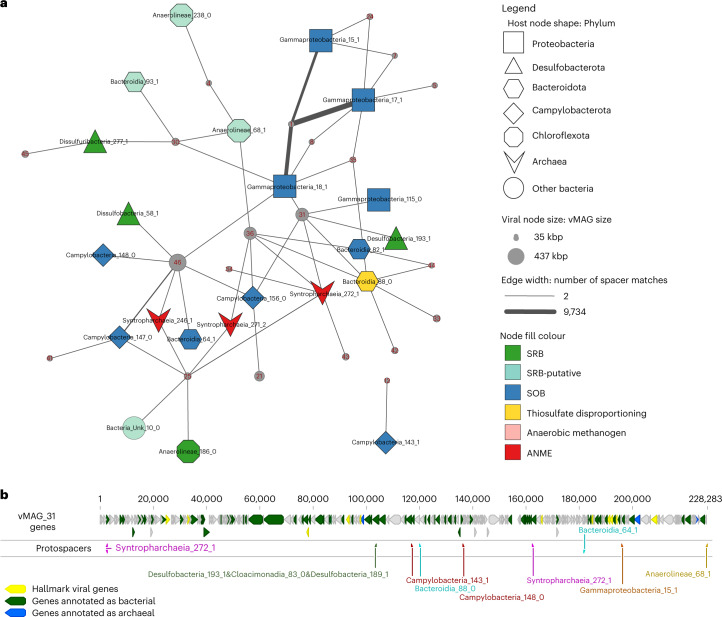
Pruned historical host-virus interactions based on CRISPR spacer-to-protospacer matches. **a**, Spacer-to-protospacer matches between rep_mMAGs and rep_vMAGs, where at least two distinct matches were found are represented with an edge. CRISPR repeats that were found in multiple rep_mMAG were excluded in this network. The edge width corresponds to the number of distinct matches. Shape and colour of host nodes denote host phylum and putative metabolisms, respectively. Size of viral nodes are scaled to the corresponding rep_vMAG length. **b**, Visualization of protospacer matches along a viral contig with spacers that are associated with CRISPRs specific to at least eight hosts belonging to different phyla and domains.

**Extended Data Fig. 5:**
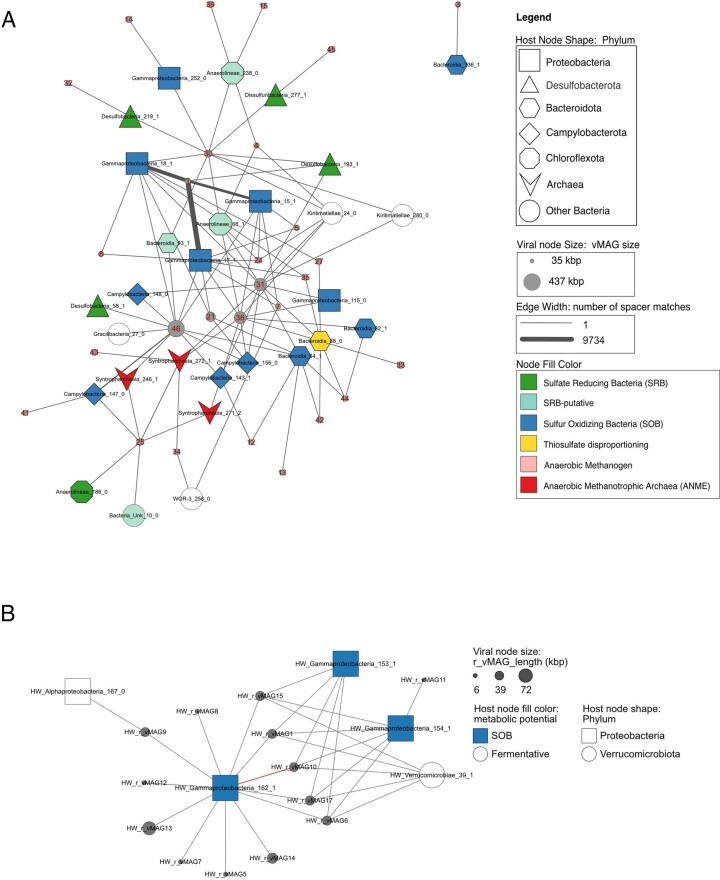
CRISPR-based immunity networks (extended). (**A**) Unpruned historical host-virus interactions based on CRISPR-spacer to protospacer matches, including host virus interactions for which only one distinct spacer-to-protospacer match was found. CRISPR repeats that were found in multiple rep_mMAG were excluded in this network. The edge width corresponds to the number of distinct matches. Color and shape of host nodes denote host phylum and putative metabolisms respectively. Size of viral nodes are scaled to the corresponding rep_vMAG length. (**B**) CRISPR-spacer to protospacer matches in hydrothermal water samples. Network was visualized using a less stringent threshold (spacer length >20 bp) than in Fig. 3 (spacer length >25 bp and each edge representing two distinct matches). Only interaction with spacer length >25 bp is highlighted with the red edge. Viral nodes are scaled to the rep_vMAG length, and rep_mMAGs with genomic capacity to carry out sulfur oxidation are colored in blue.

**Extended Data Fig. 6:**
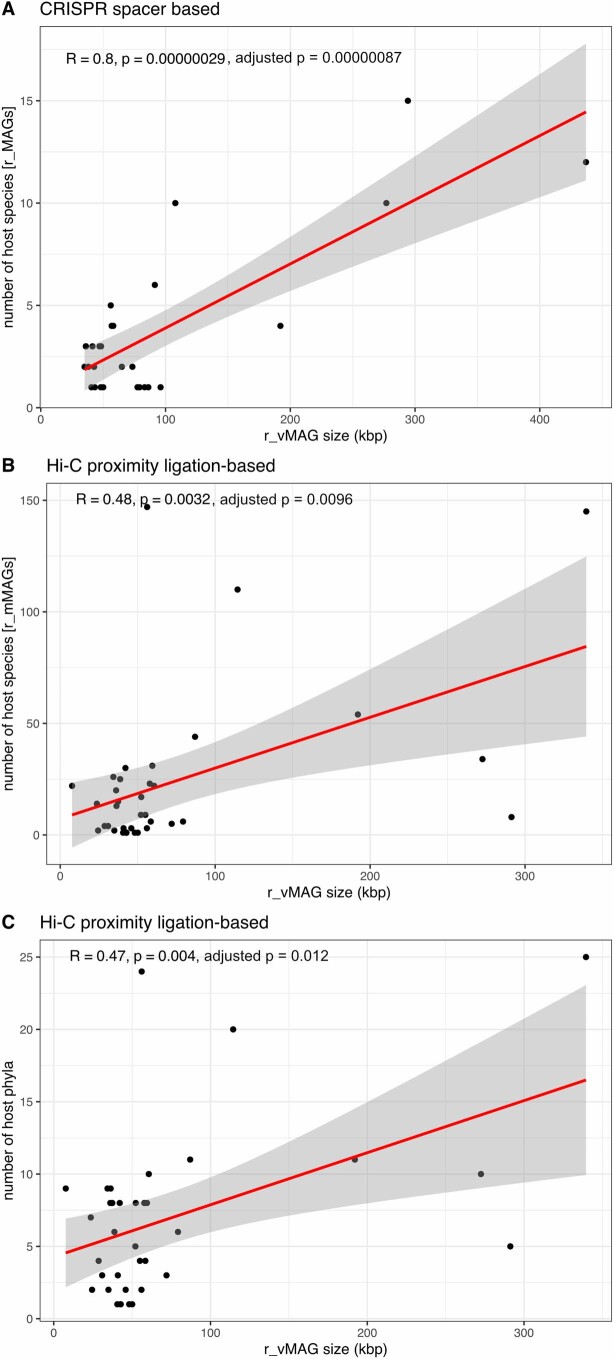
Correlations between vMAG size and ‘host range’. (**A**) Correlation between the number of hosts a rep_vMAG can be linked with using CRISPR spacer based matches and the corresponding rep_vMAG size. (**B**, **C**) Correlation between the number of hosts (**B**) and host phyla (**C**) a rep_vMAG can be linked to using Hi-C proximity ligation-based matches and the corresponding rep_vMAG size. Shaded region of error denotes 95% confidence level interval for predictions from a linear model ("lm"). Correlations were calculated using two-sided Pearson correlation test (n = 36). The p-values are multiple hypothesis corrected using bonferroni correction (k = 3).

Using the population-specific CRISPR repeats, we mined 278,929 unique spacers across the 10 metagenomes. Spacer-to-protospacer (region in the viral genome that serves as the template for the spacer and is subsequently targeted by the CRISPR-Cas system) matches between rep_mMAGs and rep_vMAGs were used to infer host adaptive immunity against specific viruses and hence, historical host-virus interactions. We identified 96 interactions between 28 rep_vMAGs and 29 rep_mMAGs resulting from 22,466 spacer-to-protospacer matches associated with 39 rep_mMAG-specific CRISPRs. A small fraction (0.01%, 25 spacers) of the protospacers were found in non-unique regions of at most 2 rep_vMAGs. The lack of high-confidence matches for the majority (66%) of CRISPRs to viral targets suggests that there may exist higher diversity in viral population than what could be detected using metagenomic sequencing, and/or that there is a rapid turnaround of viral populations in this environment. In Fig. 2a, we show CRISPR-spacer-based host-virus interactions for host-virus pairs with at least two distinct protospacer-to-spacer matches (all interactions are visualized in Extended Data Fig. 5a and are listed in Supplementary Table 8). A large majority (92%) of spacer-to-protospacer matches were between rep_vMAG_1 and 3 Gammaproteobacterial rep_mMAGs, which is consistent with rep_vMAG_1’s observed normalized abundance which is orders of magnitude higher than that of other rep_vMAGs (Fig. 1d). We observed a striking pattern of known and hypothesized syntrophic partners (ANME-SRB, SOB-SRB) with CRISPR-spacer matches to the same rep_vMAGs. Host-virus matches visualized in Fig. 2a were made using unique CRISPRs and represent immunity that probably result from historical interactions as opposed to lateral transfers of CRISPR arrays. Additionally, CRISPR-spacer matches from different microbial populations were distributed throughout the viral contig and showed no preference in targeting specific genomic regions (for example, Fig. 2b). Interestingly, we found a statistically significant positive correlation (Pearson’s two-sided correlation coefficient = 0.8, adjusted *P* < 1 × 10^−6^, *n* = 36; Extended Data Fig. 6a) between rep_vMAG size and the number of hosts they could be associated with using CRISPR spacers, suggesting that CRISPR targeting by taxonomically diverse microbes may be more common for larger viruses.

### Hi-C proximity-ligation shows host-virus genome linkages

**Fig. 3:**
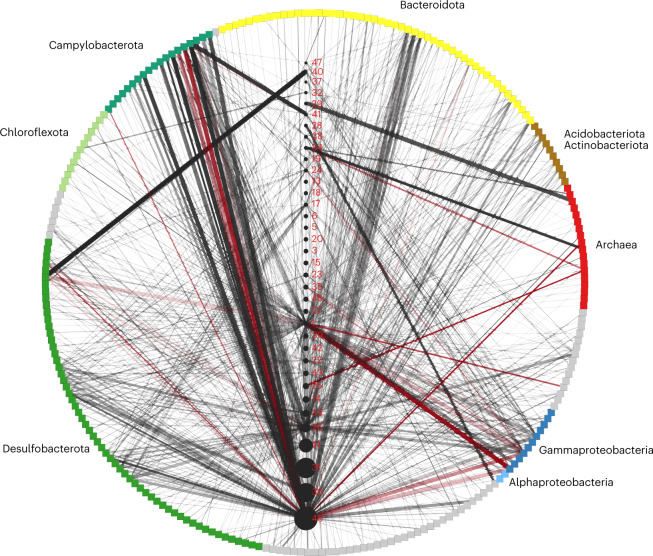
Hi-C proximity ligation informed in situ host-virus interactions network. Network visualization of rep_mMAGs and rep_vMAGs based on normalized Hi-C contacts. rep_mMAGs are positioned in a circle, in square nodes, with the colours representing taxonomic classification (grey: other). rep_vMAGs are positioned vertically in increasing rep_vMAG size in black circular nodes along the centre. rep_vMAG IDs are denoted with red labels (for example, 1 refers to rep_vMAG_1). Thickness of the edges represents the number of contig-to-contig linkages, while the darkness of the edges correlates with the maximal normalized strength of the Hi-C contacts between any two contigs in a host-virus pair. Host-virus pairs that were previously detected using CRISPR-spacer matches are coloured in red.

**Extended Data Fig. 7:**
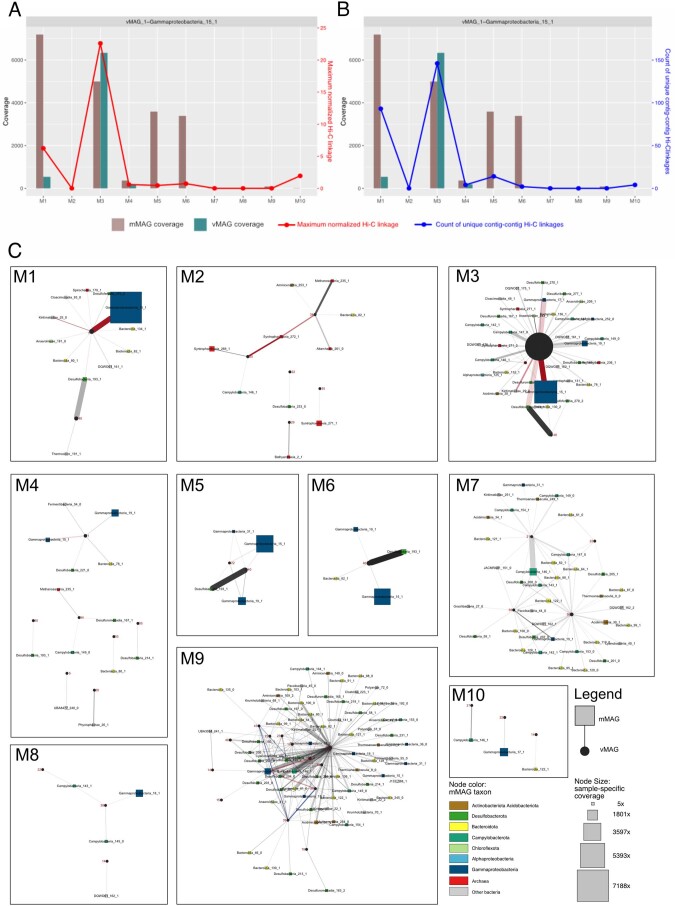
Extended Hi-C analysis. (**A**, **B**) Change in the read-mapping coverages of rep_vMAG_1 and Gammaproteobacteria_15_1 across samples. Overlaid is the intensity (A: maximum normalized Hi-C linkages between the viral and host contigs, B: count of unique Hi-C linked pairs of viral and host contigs) of Hi-C linkages between the host-virus pair across samples. (**C**) Sample-specific Hi-C proximity ligation, for host and viral MAGs for which sample-specific abundances could be reliably calculated using read mapping (coverage >5, breadth >0.7). Viral nodes (circular) are labeled according to the corresponding rep_vMAG ID. microbial nodes are colored according to the taxon, using the same color scheme as the main Fig. 3. Node sizes correspond to the sample-specific read-mapping coverages. Thickness of the edges represent the number of contig-to-contig linkages, while the darkness of the edges correlates to the maximal normalized strength of the Hi-C contacts between any two contigs in a host-virus pair. Host-virus pairs that were previously detected using CRISPR-spacer matches are colored in red. Identified Hi-C linkages between viruses are noted with blue edges.

While CRISPR spacer-to-protospacer matches provide high-confidence information on historical interactions between hosts and viruses, some hosts do not encode CRISPRs^22^ and CRISPR arrays often fail to assemble in shotgun assemblies due to their repetitive nature. In situ host-virus genome linkages can be probed using the proximity-ligation method^23^. We constructed and sequenced 10 Hi-C metagenomic libraries (totalling 1.5 billion Hi-C 150-bp read pairs; see associated statistics including per-sample mapping rate in Supplementary Table 9) that encode information on putative chromosomal contacts (see contact matrices for each sample in Supplementary Fig. 1a–j), including those between intracellular viral and host genomes. We estimated the noise-to-signal ratios of the Hi-C contacts using the binned contigs (raw noise = 0.021 ± 0.016) and order-level taxonomic classification of the binned contigs (relaxed noise = 0.016 ± 0.011; see Methods for noise ratio calculations). We detected 5,292 linkages between viral and host contigs, which could be consolidated into 859 linkages (24% of which were replicated in multiple samples) between 36 rep_vMAGs and 241 rep_mMAGs belonging to 31 different phyla, revealing a highly nested network of potential interactions between hosts and viruses (Fig. 3; all interactions are listed in Supplementary Table 10). After normalization^24^, we observed that some host-virus genome interactions were more pronounced in both the count of unique linkages identified between host and viral contigs (visualized as width of edges in Fig. 3) and the maximum strength of linkages (maximum count of normalized contacts between a pair of viral and microbial contigs; visualized by darkness of edges) between a host-virus pair. These more pronounced host-virus linkages can be interpreted as signals for the presence of in situ infections between the host-virus pair, where Hi-C reads could capture viral genomes actively replicating inside certain host cells. In some cases, we can align the strong proximity-ligation signal between rep_vMAG_1 and Gammaproteobacteria_15_1 in sample M3 with increased ratios between vMAG and mMAG coverages (Extended Data Fig. 7a) and orders of magnitude higher rep_vMAG relative abundances in this sample (Fig. 1d). However, it is important to note that for most other host-virus pairs, these measures of significant Hi-C linkages do not necessarily correlate with higher rates of population-wide infection, as the Hi-C capture of infection events at the time of crosslinking is relatively rare. Here we use these signals to identify potential primary hosts of viruses and decompose their polyvalent interactions. For instance, we observed consistent patterns across samples where viruses interact with multiple hosts, but with more significant interactions with a subset of hosts, regardless of the high variability in both viral and microbial abundances between samples (Extended Data Fig. 7b). We observed Hi-C linkages overlapping with CRISPR-spacer matches in a number of host-virus population pairs (visualized as red edges), probably reflecting within-population and/or strain-level heterogeneity in CRISPR-based immunity^25^ (where different subsets/strains of host population possess CRISPR immunity against different viruses). Our host-virus interaction network based on Hi-C linkages are consistent with what we have observed using CRISPR-based approaches, with viruses co-localizing with phylogenetically distant organisms featuring interdependent metabolisms. Hi-C linkages in metagenomes contain inherent noise, therefore we cannot reject the possibility that some of the inferred host-virus linkages may be false positives. Nevertheless, consistent results between CRISPR and proximity-ligation data suggest that the virus-microbe interaction network is more nested in this hydrothermal mat than typically observed or expected. We observed a pattern where larger rep_vMAGs (viral node size) exhibit more numerous (more edges) and significant (thicker and darker edges) linkages with phylogenetically diverse rep_mMAGs, similar to the pattern observed in CRISPR data (Extended Data Fig. 6). However, there exists an explicit bias towards contig length and coverage on Hi-C read signal that cannot be fully controlled for even after normalization^24^; thus, this observation needs further examination using complementary methods less biased towards contig length (for example, single-cell viral tagging^26^). Interestingly, we found no correlation between the nucleotide diversity, average abundances or habitat range of rep_vMAGs and their host ranges inferred by CRISPR-based and Hi-C based methods.

### Comparison with hydrothermal plume water samples

**Extended Data Fig. 8:**
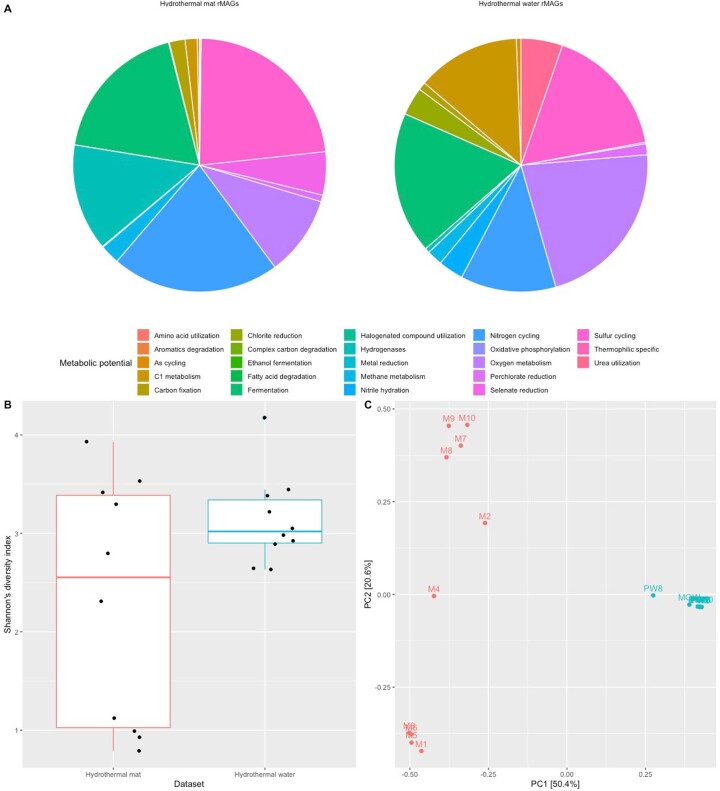
Comparison between ten hydrothermal mat metagenomes and ten hydrothermal water metagenomes. (**A**) Metabolic gene content annotated and categorized using METABOLIC in binned rep_mMAGs from the two metagenomes. (**B**) Shannon diversity indices between the two sample sets. No statistically significant differences were detected (Welch’s t-test, n = 20 biologically independent samples, two-sided, p > 0.05). Box plot shows the quartiles (25, 50, 75 percentiles) with the upper and lower whiskers showing the max and min value within 1.5 times the interquartile respectively. (**C**) Principal coordinate analyses of the rep_mMAGs in the two datasets; hydrothermal mat samples are colored in red and hydrothermal water samples are colored in blue. The percentage of variance explained by each axis is shown in the axis label.

**Extended Data Fig. 9:**
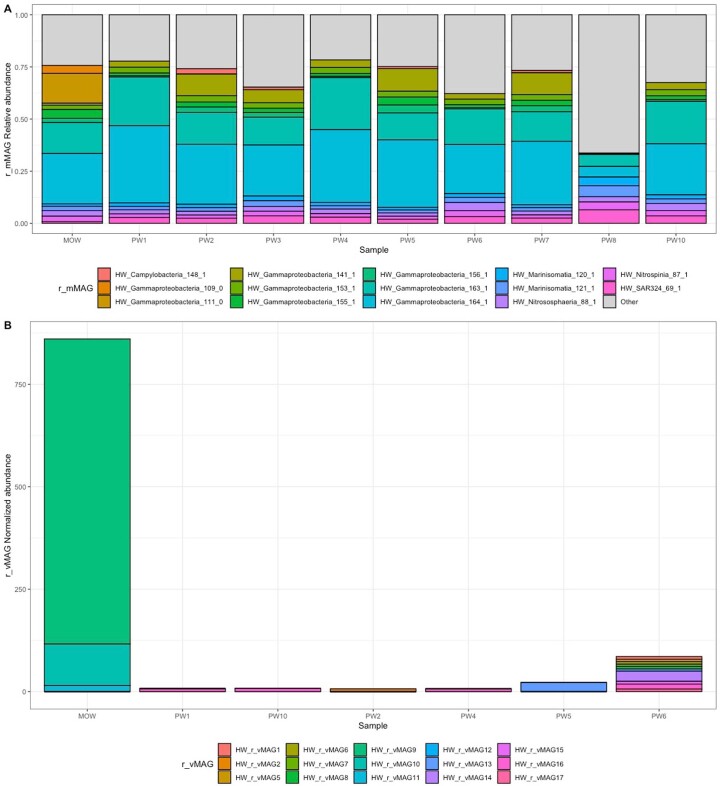
Microbial and viral composition of the hydrothermal water samples. (**A**) Relative abundances of top ten more abundant rep_mMAGs from hydrothermal waters samples. (**B**) Normalized abundances of high quality and complete rep_vMAGs in hydrothermal water samples. Only rep_vMAGs detected at >5 coverage and >0.7 breadth using read mapping are shown and proviruses are excluded.

We posited that the density and the spatial structure of the microbial mat contributed to the nested patterns of the CRISPR-based immunity network. To explore this relationship, we conducted the same CRISPR-based host-virus network analysis on 10 metagenomes of hydrothermally influenced water samples (hereafter referred to as hydrothermal water (HW) samples; for sample descriptions, see Supplementary Table 1b) consisting of 9 samples from a nearby hydrothermal plume (~45 km away from the mat) and 1 sample from the water overlying the sampled mat. The HW metagenomes were similar in both sequencing depth and assembly size to the mat metagenomes (Supplementary Table 1c; Welch’s *t*-test, two-sided, *n* = 20, *P* > 0.05). We binned 168 mid- to high-quality rep_mMAGs (see Supplementary Table 11 for the full description) across the 10 HW assemblies, and although taxonomically distinct from the rep_mMAGs recovered from the mat assemblies, the two datasets featured similar metabolic capabilities (Supplementary Table 12 and Extended Data Fig. 8a) and similar levels of species evenness (Extended Data Fig. 8b, Welch’s *t*-test, two-sided, *n* = 20, *P* > 0.05). The microbial communities of the HW samples were more homogeneous than the mat samples (Extended Data Fig. 8c) despite the larger physical distances between the HW samples. Similar to the mat samples, HW samples were dominated by two sulfur oxidizing Gammaproteobacteria (HW_Gammaproteobacteria_164_1, HW_Gammaproteobacteria_163_1; Extended Data Fig. 9a). Interestingly, we observed an order of magnitude less frequent detection of CRISPR loci in the HW assemblies compared with the mat assemblies (Supplementary Table 13, Welch’s *t*-test, two-sided, *n* = 20, *P* = 0.001). Furthermore, only 12 of the CRISPRs in the HW assemblies could be associated with medium- to high-quality MAGs (Supplementary Table 14), resulting in a much sparser and less robust CRISPR-based immunity network (Extended Data Fig. 5b and Supplementary Table 15), with only one confident interaction between an SOB (HW_Gammaproteobacterira_162_1) and a virus. The similarities between the plume and mat samples, such as geographical proximity, community metabolic capabilities and sequencing depth, provide a rationale and opportunity for comparison. Lower abundances of the CRISPRs in the plume samples indicate that the plume communities are less reliant on CRISPR-based adaptive immunity. The transferability and specificity of CRISPR-based immunity confer ecological significance to this observation, raising the question of how such immunological memory is selected for in different environments. While this comparison illuminates key differences in the nature and extent of host-virus interactions between the mat and the plume, there are some caveats to consider for further interpretation: first, the sparseness in the plume CRISPR-based immunity network is likely due in part to the lower abundance and diversity of recovered viral contigs (Supplementary Table 16 and Extended Data Fig. 9b), where only the fraction of viruses that were infecting microbes and/or were attached to particles larger than 0.022 µm were recovered. Second, differences in the CRISPR-based immunity do not necessarily reflect the patterns of the underlying networks of in situ host and virus interactions.

### Global distribution of microbial domain-crossing viruses

To characterize the prevalence of host-viral interactions across large phylogenetic distances, we looked for viruses that map to spacers found in both archaeal and bacterial CRISPR loci in public databases. We detected 26 viruses across 25 samples originating from 5 sites (Table 1). These viruses were found in ecosystems where biofilm formation has been evidenced (for example, anaerobic digester sludges^27^, petrochemical wastewater in a tailings pond^28^ and a CO_2_-rich subsurface aquifer^29^) and metabolic interdependencies have been highlighted using various methods such as co-occurrence networks^30^, metatrascriptomics^31^ and lipidomics^29^. Additionally, many of the matching CRISPR spacers were found in known or hypothesized syntrophic taxa, such as Methanosarcinales^32^, *Methanoculleus*^33^, *Smithella*^34^ and Thermotogales^35^ (Supplementary Table 17). As mentioned above, CRISPR-based host-virus interaction inference is limited to environments where abundant CRISPR loci can be assembled and binned. Therefore, it is possible that these host-virus interactions across large phylogenetic distances may be more common and more widespread in nature than can be detected using this method. For instance, we detected very few binned high-confidence (Methods) CRISPR loci in MAGs from large metagenome datasets that lacked microbial mats and featured lower microbial density (and possibly fewer metabolic syntrophies), such as those in oligotrophic water samples from the Hawaii Ocean Time Series and the Bermuda Atlantic Time Series^36^, as well as those in more canonical deep-sea sediments collected off the coast of San Francisco^37^ (Supplementary Table 13).

**Table 1:** Publicly available metagenomes with viruses matching both bacterial and archaeal CRISPR spacers

Sample type	Location	Latitude, longitude	No. of viruses matching bacterial and archaeal CRISPRs	Bacterial host	Archaeal host	Total assembly size (Gbp)	IMG genome ID
Petrochemical wastewater pond	Alberta, Canada	57.12167391, −111.6126031	2	Anaerolineales	Methanosarcinales	7.4	3300002446, 3300002821
Groundwater geyser	Utah, USA	38.95178543, −110.1358936	14	Hydrogenophilales*,* Proteobacteria*, Galllionella*	Micrarchaeota	9.1	3300005236, 3300025150, 3300025142, 3300025833, 3300025007, 3300025126, 3300025035, 3300025034, 3300025839, 3300025129, 3300025845, 3300025139, 3300025032
Anaerobic digester	Wisconsin, USA	43.96538753, −88.08366106	1	Thermotogales	*Mathanoculleus,* Methanomicrobiales	0.3	3300028628
Anaerobic digester	Waagenigen, Netherlands	51.98641046, 5.665628909	8	Anaerolineales	Methanosarcinales	4.3	3300033177, 3300033170, 3300033174, 3300033169, 3300033176, 3300033172, 3300033178, 3300033175
Anaerobic digester	Oakland, USA	37.80409292, −122.2708158	1	*Smithella*	*Mathanoculleus*	0.4	3300025657
Deep-sea hydrothermal microbial mat	Guaymas Basin, Mexico	27.00647127, −111.4093484	5	Gammaproteobacteria*, Desulfobacteria, Campylobacteria,* Kritimatiellae*,* Bacteroidia*, WOR-3,* Gracilibacteria	‘*Ca*. Syntropharchaeia’ (ANME-1)	6.1	This study

Statistics from this study are shown in the last row for comparison.

### Selection and diversification of microbial mat genes

**Extended Data Fig. 10:**
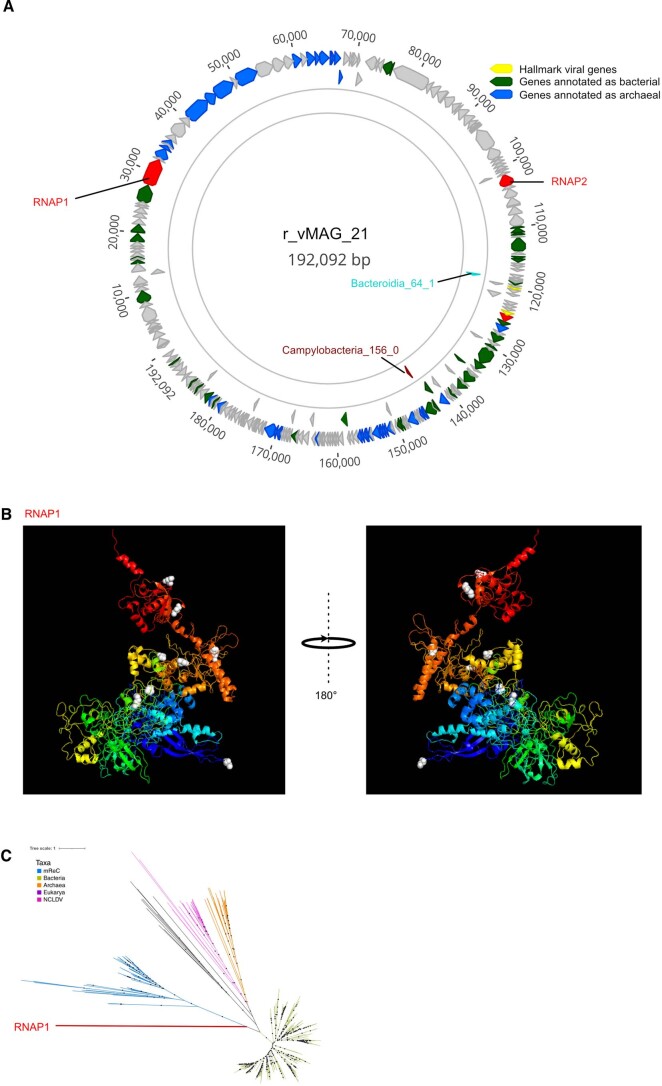
Viral DNA-directed RNA-polymerase subunit (RNAP) undergoing diversifying selection. (**A**) Genomic context of RNAP1 undergoing selection. Note the presence of a non-homologous RNAP gene encoding beta subunit found in the same viral genome. (**B**) Predicted structure and locations of non-synonymous polymorphisms (visualized with white sphere). (**C**) Placement of the RNAP1 sequence in the tree previously published by Weinheimer and Aylward (2020), where it distantly clusters with sequences from mReC (multimeric RNAP-encoding Caudovirales). Branches strongly supported with at least 95 for ultrafast bootstrap are marked with black circles.

On the basis of the highly nested nature of the host-virus interaction network and the high heterogeneity in the viral community between the mat samples, we hypothesized that many of the genes undergoing selection in both viruses and microbes would be associated with host range and viral defence, respectively. We calculated pN/pS ratios (ratio of non-synonymous to synonymous polymorphisms) for viral and microbial genes and attempted to predict their functions. We identified 18 viral genes putatively undergoing diversifying selection (pN/pS > 2.5); however, most could not be annotated with a function. Interestingly, 3 of the 4 annotated genes undergoing diversifying selection were involved in DNA and RNA metabolism, such as genes encoding DNA-directed RNA polymerase (RNAP) beta and beta prime (rep_vMAG_21), DNA ligase (rep_vMAG_31) and Superfamily II DNA/RNA helicase (rep_vMAG_6). We also detected a LamG domain-containing protein (vMAG_4), possibly involved in signalling and cell adhesion, to be undergoing diversifying selection. The gene encoding RNAP in rep_vMAG_21 (RNAP1; Extended Data Fig. 10a) featured the highest pN/pS ratio of 4.9, with 8 non-synonymous mutations scattered throughout the protein (Extended Data Fig. 10b). Notably, rep_vMAG_21 featured a second RNAP gene fragment encoding the beta subunit (RNAP2) (Extended Data Fig. 10a) that is not homologous to RNAP1 and not seemingly undergoing selection, possibly contributing to the relaxation of purifying selection on RNAP1. RNAP1 was highly divergent from the previously characterized RNAP sequences and was rooted at the base of the *Caudoviricetes* multimeric RNAP clade^38^ (Extended Data Fig. 10c). This example of diversifying selection on RNAP1 suggests that these viruses may play an important role in expediting the evolution of housekeeping proteins that typically undergo purifying selection in cellular organisms. Microbial genes undergoing diversifying selection (pN/pS > *2*) included genes encoding products involved in various defence systems, such as type II toxin-antitoxin system RelE/ParE toxin, HindIII family type II restriction endonuclease, Type III-B CRISPR module RAMP protein Cmr1, as well as genes involved in more recently characterized PARIS and Septu anti-phage arsenal^39^.

## Discussion

**Fig. 4:**
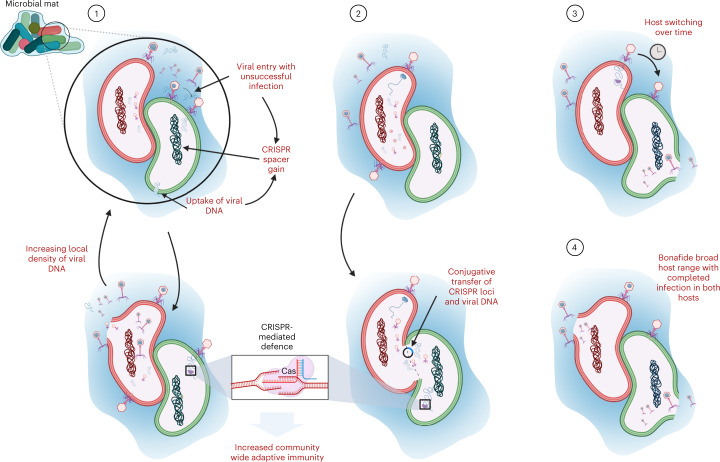
Four proposed models for host-virus interactions in ecosystems with high microbial density and metabolic interdependence. Red and green cells represent phylogenetically distant and metabolically independent hosts (for example, ANME-SRB). Blue shading represents an EPS matrix that limits diffusion of viral and extracellular DNA. In the first model, we illustrate the possibility of ‘promiscuous’ viral adsorption and entry into a non-primary host cell (green), which results in a CRISPR-spacer gain event. Alternatively, the limited dispersal potential due to the EPS may result in an increased local density of viral particles and viral DNA following a lysis event of the primary host (red). Consequently, this can lead to a higher likelihood of a non-primary host cell’s natural uptake of viral DNA, also resulting in a spacer gain event. In the second model, we present the possibility of contact-based transfer of CRISPR arrays and viral DNA. This would also result in a gain of a CRISPR-spacer event by a non-primary host cell (green). In both models, this results in CRISPR-mediated augmented community-wide immunological memory and resilience. In the third model, we present the possibility of viral host switching over time, from primary host (red) at *T* = 0 to its nearest syntrophic partner (green) as the initial host evolves against the virus. Finally, in the last model, we consider the possibility of a bonafide broad host range with successful viral infection in both hosts.

In this study, we investigated how microbial density and metabolic interdependence shape host-virus interactions in a microbial mat. Our results taken together provide compelling evidence that viruses probably interact with phylogenetically distant microbes in microbial mats and biofilms that feature high biomass density, diversity and metabolic cooperation. We propose four non-mutually exclusive models to better contextualize and provide potential explanations for this unexpected observation (Fig. 4). In the first model, we propose that the viral genome may enter cells that the virus cannot infect (that is, a ‘non-primary host’) in ecosystems where viral DNA and particles remain in high proximity to a dense, diverse community that is maintained in part due to syntrophies and the EPS matrix. The second model presents the possibility of contact-based transfer of viral particles and/or genomes between or among syntrophic microbes, even those in different domains. Conjugative transfers across large phylogenetic distances have been evidenced^40^ and are hypothesized to be more common in nature^41^, and our data support this supposition. In both cases, the introduction of a viral genome to a non-primary host cell would trigger CRISPR-based immune responses and result in a gain of spacer events^42^. This mechanism could lead to an increased immunological memory and response against phages across populations and may thus be particularly selected for when the fitness of an organism is tightly linked to the resistance of its syntrophic partner against phages. This expands upon the concept of within-population pan-immunity^43^ to possibly include shared immunity across populations and large phylogenetic distances. In particular, this highlights the underexplored linkage between metabolic symbiosis and ‘defensive symbiosis’^44^ among microbes. In the third and fourth models, we propose the possibility that high-density ecosystems such as microbial mats are hotspots for viral host switching and/or host-range expansion. However, such changes and/or expansion in host range of individual phages remain to be confirmed experimentally (that is, through evidence of virion production).

These interactions between viruses and non-primary hosts have broad ecological and evolutionary implications, including but not limited to: (1) mediation of horizontal gene transfer across domains and phyla, (2) increased selective advantage of adaptive immunity in ecosystems featuring high microbial density and syntrophy and (3) diversification of both microbial and viral genes involved in defence and host-range expansion, respectively. Our findings caution against relying solely on CRISPR-based methods for inferring viral infectivity of hosts because non-infection interactions can also lead to CRISPR-spacer gain events. Our findings also highlight the potential for using CRISPR-spacer information to explore host-virus interactions beyond infection, such as untangling horizontal gene transfer networks across large phylogenetic distances and characterizing community-wide collaborative immunity. Furthermore, we propose that such nested ‘immunity networks’ can be used to generate hypotheses on novel microbe-microbe interactions (for example, syntrophy). Fundamentally, these expanded models of host-virus interactions present an important dimension to consider as we elucidate the underlying mechanisms of coexistence, competition and cooperation in high-density ecosystems.

## Methods

### Hydrothermal mat sample collection and metagenomic and Hi-C library sequencing

Microbial mat samples were collected during a research expedition (RR2107) on R/V *Roger Revelle* to the southern Guaymas Basin using the remotely operated vehicle *Jason* on dive J2-1398 on 28 November 2021. A Marine Science Research permit (Autorizacion EG0072021) was issued by the Mexican National Institute of Statistics and Geography on 21 July 2021 for the sample collection and scientific activities in the fieldwork location. Ten pushcore samples were taken across a ~10 m wide microbial mat at coordinates 27.00647191° N and 111.40935798° W at a water depth of 2,005.3 m (Fig. 1a,b). The sampled microbial mat could be visually delineated by the distinct white, yellow and orange patchy colorations characteristic of Beggiatoa mats^13^. A total of 10 pushcores (7.5 cm in diameter) were inserted (~25 cm below seafloor) within the centre of the microbial mat ~60–70 cm from each other. Immediately after sediments were sampled, the sampling wand aboard ROV *Jason* was inserted into the sediment adjacent to the pushcore scar to probe the temperature at ~7 cm below seafloor. After recovery, pushcores were processed immediately in the on-board laboratory at room temperature. The top layers primarily composed of microbial mat were subsampled using sterile metal spoons into 10 ml cryovials, which were immediately flash-frozen in liquid nitrogen and stored at −80 °C until further processing. Bulk DNA extraction was performed using ZymoBIOMICS DNA Miniprep kit (cD4300; Zymo Research) according to the manufacturer’s instructions. Hi-C and shotgun libraries were prepared for each sample using the ProxiMeta service of Phase Genomics. Hi-C libraries were generated using restriction enzymes Sau3AI and MluCI^45^. Hi-C and shotgun libraries were sequenced on a single lane of an Illumina NovaSeq S4 system (paired-end, 150 bp) .

### Geochemical analysis of hydrothermal mat samples

Before sampling the microbial mat and sediments within the pushcore, 1 ml of seawater overlying the sediment was added to 1 ml of 5% zinc acetate solution. For sediment porewater geochemistry analysis, the top 0–2 cm depth interval from each sediment pushcore was subsampled immediately after the overlying microbial mat was removed. The sediment was sampled with a stainless-steel spoon into argon-flushed 50 ml plastic centrifuge tubes while under a constant flow of argon over the sediment to minimize oxidation of oxygen-sensitive solutes. The sediment samples were centrifuged at 3,400 × *g* for 10 min to separate the porewater from the solid phase. One ml of porewater was added to 1 ml of 5% zinc acetate solution and immediately frozen at −20 °C for future analysis of dissolved sulfide in the laboratory. Two ml of the remaining seawater and porewater was transferred into a 2 ml cryovial and frozen at −20 °C for future analysis of dissolved sulfate concentrations. Preserved seawater and porewater were processed within 1 yr of collection by the Treude research group at the University of California, Los Angeles. To determine dissolved sulfide concentrations, seawater and porewater were analysed according to a previously published method^46^. Sulfide concentrations were determined using a Shimadzu UV spectrophotometer (UV-1800). Seawater and porewater not preserved with zinc acetate were analysed for sulfate concentrations using an ion chromatograph (Metrohm 761)^47^.

### Hydrothermally influenced water sample collection and metagenomic sequencing

Eight plume water (PW1–PW8) samples were collected during the same research expedition as the mat samples, near a pre-identified hydrothermal vent source (27.40921631° N, 111.38910334° W, water depth 1,810 m) using a conductivity–temperature–depth (CTD)-rosette system (Sea-Bird) fitted with 24 10-l-capacity Niskin bottles, between 17–18 November 2021. PW10 and mat overlying water samples were taken using the 5-l-capacity Niskin bottle on the ROV *Jason* near the source of the hydrothermal activity (19 November 2021) and above the sampled hydrothermal mat (29 November 2021), respectively. Detailed sample information including depth and coordinates can be found in Supplementary Table 10. Upon CTD recovery, Niskin bottles were emptied into pre-washed (1× 10% HCl wash, 2× milli-Q wash, 1× sample water wash) cubitainers, which were then stored at 4 °C until filtration. Filtration was done on board using a peristaltic pump (SN 2021291435, ProMinent) at a rate of 10 l h^−1^. Inline pre-filters of 130 µm and 15 µm (8991T31, McMaster-Carr) were used before final filtration onto 0.22 µm PES membrane Sterivex filters (SVGP01050m, MilliporeSigma), which were subsequently stored at −80 °C. Between samples, tubing and pre-filters were washed using 10% HCl, Milli-Q and sample water flushes. Genomic DNA was extracted from a quarter of Sterivex filters using DNeasy PowerWater kit (14900-100-N, Qiagen) and sequenced on the Illumina NovaSeq S4 system (paired-end, 150 bp) at the Bauer Core Facility at Harvard University.

### Host genome binning, annotation and taxonomic classification

Shotgun reads were quality filtered using BBduk (https://sourceforge.net/projects/bbmap/) and sickle (https://github.com/najoshi/sickle), and assembled using metaSPAdes v3.15 (ref. ^48^). Bacterial and archaeal MAGs were binned by consolidating results from multiple binning tools (maxbin2 v2.2.7 (ref. ^49^), metabat2 v2.15 (ref. ^50^), CONCOCT v1.1.0 (ref. ^51^), ABAWACA v1 (https://github.com/CK7/abawaca) and ProxiMeta Hi-C deconvolution^45^) using DAS Tool^52^. Quality of the MAGs was estimated using CheckM v1.1.3 (ref. ^53^) and only medium- to high-quality (>70% completeness and <10% contamination) MAGs were used for subsequent analysis. Mid- to high-quality MAGs were dereplicated at 97% ANI using dRep v3.0.1 (ref. ^54^) and were designated as representative MAGs (rep_mMAGs). rep_mMAGs were taxonomically classified using GTDB-Tk v1.7.0 (ref. ^55^). Genes were predicted using Prodigal v2.6.3 (ref. ^56^) and annotated by aligning them using Diamond v2.0.7.145 (ref. ^57^) against the UniRef100 database^58^ with an *e*-value cut-off 1 × 10^−5^. Additionally, METABOLIC v4 (ref. ^59^) and DefenseFinder v1 (ref. ^60^) were used to identify potential metabolic and antiviral genes, respectively.

### Viral scaffold prediction, viral genome binning and annotation

Viral scaffolds were predicted using VirSorter2 (ref. ^61^) and VIBRANT v1.2.1 (ref. ^62^) from assembled scaffolds larger than 1 kb in length; the union set of the output were used for viral MAG (rep_vMAG) binning using vRhyme v1.1.0 (ref. ^63^) after dereplication using CD-HIT^64^ at 95% sequence identity and 85% alignment coverage^65^ and mapping reads using Bowtie2 v2.3.2 in sensitive mode^66^ for each sample. Viral scaffolds were taxonomically classified using vConTACT v2^20^. Circular sequences as identified by vRhyme were added to the final rep_vMAG set, which were subsequently quality checked using CheckV v0.9.0 (ref. ^18^). Only rep_vMAGs predicted to be ‘high-quality’ or ‘complete-quality’ (hereafter referred to as high- to complete-quality) using high-confidence prediction methods (‘AAI-based’, ‘DTR’, ‘ITR’) were kept for further analyses. Genes were predicted and annotated using Prokka v1.14.6 (ref. ^67^) from high-quality rep_vMAGs by aligning them against the UniRef100 database^58^, with an *e*-value cut-off 1 × 10^−6^. Genes were also annotated using MMseqs2 v13.5 (ref. ^68^), Diamond v2.0.15 (ref. ^57^) and HMMER v3.3.2 (ref. ^69^) by aligning them against PHROGS v4 (ref. ^70^), COG-20 (ref. ^71^) and VOG v213 (ref. ^72^) databases, respectively. DRAM-v v1.3.5 (ref. ^73^) was used to identify candidate AMGs in rep_vMAGs. Genes with AMGs score of 1–3 and AMG flag of -M and -F were classified as candidate AMGs, then their position in the viral contig as well as the functional annotation of candidate AMGs and their neighbouring genes were manually checked.

### CRISPR-Cas analysis, spacer extraction and protospacer-to-spacer matching for immunity network

CRISPR-Cas loci were identified and *cas* genes were subtyped from all medium- to high-quality mMAGs using CRISPRCasFinder v4.2.20 (ref. ^74^) and DefenseFinder v1 (ref. ^60^). Only repeats from CRISPR arrays with evidence level 4 were extracted for CRISPR spacers from quality filtered shotgun reads using metaCRAST^75^. Local alignments of extracted spacers with lengths greater than 25 bp against high- to complete-quality vMAGs (all unique high- to complete-quality viral MAGs before dereplication at 95% sequence identity and 85% alignment coverage^65^) were searched using ‘blastn-short’^76^. Only BLAST matches with 100% alignment coverage and at most two mismatches were considered as high-confidence protospacer-to-spacer matches. CRISPRs that were associated with more than one population (rep_mMAG) were excluded from the immunity network as the extracted spacers from shared repeats cannot be reliably assigned to a specific taxon. The host-virus network was visualized using Cytoscape v3.9.1 (ref. ^77^).

### Hi-C proximity-ligation-based host-virus matching

Hi-C chimaeric reads were quality filtered using BBduk and sickle, and mapped using BWA mem v0.7.17 with flag -5SP against a combined scaffold database of rep_mMAGs and rep_vMAGs. Before read-mapping, we removed redundancies in the database by dereplicating the scaffolds using CD-HIT-EST with flags -aS 0.85 -c 0.95 to prevent Hi-C reads mapping across very similar scaffolds resulting in false positive host-virus matches. Scaffold coverages were calculated by mapping metagenomic shotgun reads using bbmap (sourceforge.net/projects/bbmap/) against the same database. Hi-C contact maps for each sample were normalized using the unlabelled version of HiCZin^24^. The host-virus infection network was visualized using Cytoscape v3.9.1 (ref. ^77^). Noise-to-signal ratios were calculated using two methods: (1) raw noise: (# inter-mMAG contacts)/(# intra-mMAG contacts) and (2) relaxed noise: (# inter-order contacts)/(# intra-order contacts), where # inter-mMAG contacts = # Hi-C read pairs mapping to different mMAGs, # intra-mMAG contacts = # Hi-C read pairs mapping to the same mMAG or contig, # inter-order contacts = # Hi-C read pairs mapping to different mMAGs belonging to different taxonomic orders, # intra-order contacts = # Hi-C read pairs mapping to the same contig or contigs binned to the same taxonomic order. Log-transformed contact matrices were visualized using a modified bin3C^78^ mkmap function with flag max_image_size = 5,000.

### Identification of viruses matching both archaea and bacterial CRISPR spacers in public datasets

To evaluate how frequently individual virus genomes are matched to both archaea and bacteria CRISPR spacers, we leveraged the IMG/VR v3 online database^79^, which includes 47,513 genomes linked to a bacterial or an archaeal CRISPR spacer. Among these, we collected the list of genomes that showed matches to both bacterial and archaeal CRISPR spacers (*n* = 26). Sample location and ecosystem type were obtained from the Gold database^80^.

### CRISPR loci detection in other environments

CRISPR repeats were identified using CRISPRCasFinder v4.2.20 (ref. ^74^) from 891 medium- to high-quality MAGs^81^ from HOT and BATS metagenome time series^36^ and 209 medium- to high-quality MAGs from deep-sea sediments (36.61° N, 123.38° W, water depth 3,535 m) 115 km off the coast of San Fransisco^37^.

### SNV calling, nucleotide diversity, pN/pS and abundance calculation for rep_mMAGs and rep_vMAGs

Reads were mapped to combined rep_mMAG and rep_vMAG databases using Bowtie2 in sensitive mode. Read-mapping-based SNV calling and subsequent population genetics analyses were conducted using inStrain v1.3.1 (ref. ^82^) using default settings, except for minimum percent identity filtering at 94%. For rep_mMAGs with an average coverage of >5× and breadth (fraction of the rep_mMAG covered by at least one read) of >0.7, relative abundances in each sample were determined using the genome-wide average read-mapping coverage. For rep_vMAGs with an average coverage of >5× and breadth >0.7, normalized abundances in each sample were calculated by normalizing the average coverage of viral scaffolds in each rep_vMAG by the number of reads in each sample.

### Visualization

All graphs were visualized using ggplot2 v3.3.6 (ref. ^83^) in R v4.0.2.

### Reporting summary

Further information on research design is available in the Nature Portfolio Reporting Summary linked to this article.

## Supplementary information

Supplementary Information Supplementary Fig. 1a–j.

Reporting Summary

Supplementary Tables Supplementary Table 1. Sample descriptions and data accessions. **a**, Description of the ten deep-sea hydrothermal mat samples. **b**, Description of the ten hydrothermal water samples. **c**, Statistics for shotgun assemblies. Supplementary Table 2. Statistics of the rep_mMAGs binned across the ten mat samples. Supplementary Table 3. Genome-based metabolic capabilities and other genetic features of rep_mMAGs in the mat. Supplementary Table 4. Information on the high-quality and complete rep_vMAGs binned across the ten mat samples. Supplementary Table 5. Annotations of the high-quality and complete rep_vMAGs. Putative AMGs are flagged. Supplementary Table 6. List of rep_mMAGs with binned Cas loci. Identification and subtyping of Cas loci were conducted using DefenseFinder. Supplementary Table 7. List of binned CRISPR repeats and their associated populations (rep_mMAGs). Supplementary Table 8. All CRISPR-spacer to protospacer matches in the mat with spacer length >25 with at most two mismatches. Supplementary Table 9. Hi-C library statistics. Supplementary Table 10. Hi-C normalized linkage between rep_vMAG and rep_mMAG. Contig-to-contig linkage information was consolidated by count of linkage, average residual (normalized ‘strength’) of the linkage and maximum residual of the linkage. Supplementary Table 11. Statistics of the rep_mMAGs binned across the ten hydrothermal water samples. Supplementary Table 12. Genome-based metabolic capabilities and other genetic features of rep_mMAGs in the hydrothermal water samples. Supplementary Table 13. Number of high-confidence (evidence level = 4) CRISPR repeats binned across environments. For hydrothermal mat samples and hydrothermal mat samples from this study, we include information on the total number of evidence level 4 CRISPRs detected across environments as well as those that were binned in mid- to high-quality MAGs. Supplementary Table 14. Binned CRISPR repeats in hydorthermal water samples. Supplementary Table 15. All CRISPR-spacer to protospacer matches in hydrothermal water samples with spacer length >20 with at most two mismatches. Supplementary Table 16. Information of the high-quality and complete rep_vMAGs binned across the ten hydorthermal water samples. Supplementary Table 17. List of UViG ID and their putative hosts and host-prediction methods.

## Data Availability

Sequence data (including raw sequences, assemblies, rep_mMAG and rep_vMAGs) investigated in this study were deposited to NCBI under BioProjects PRJNA879229 (mat samples) and PRJNA879230 (HW samples). SRA accession numbers are available in Supplementary Table 1c (shotgun libraries) and Supplementary Table 9 (Hi-C libraries), and BioSample IDs are listed for rep_mMAGs in Supplementary Tables 2 and 11, and for rep_vMAGs in Supplementary Tables 4 and 16. The UniRef100 database is accessible at https://www.uniprot.org/help/downloads, the IMG/VR database at https://img.jgi.doe.gov/vr and the GOLD database at https://gold.jgi.doe.gov/. PHROGs (https://phrogs.lmge.uca.fr/), COG-20 (https://www.ncbi.nlm.nih.gov/research/cog-project/) and VOG (https://vogdb.org/) databases are available online.
