# Supplementary material for: Viruses interact with hosts that span distantly related microbial domains in dense hydrothermal mats
Source: Nat Microbiol. 2023 Apr 6;8(5):946–57. doi: 10.1038/s41564-023-01347-5 (PMC10159854; doi:10.1038/s41564-023-01347-5)
Supplement: Supplementary file 1 — Supplementary Fig. 1a–j. [file 41564_2023_1347_MOESM1_ESM.pdf]

nature microbiology

Article https://doi.org/10.1038/s41564-023-01347-5

# Viruses interact with hosts that span distantly related microbial domains in dense hydrothermal mats

In the format provided by the authors and unedited

**A. M1**

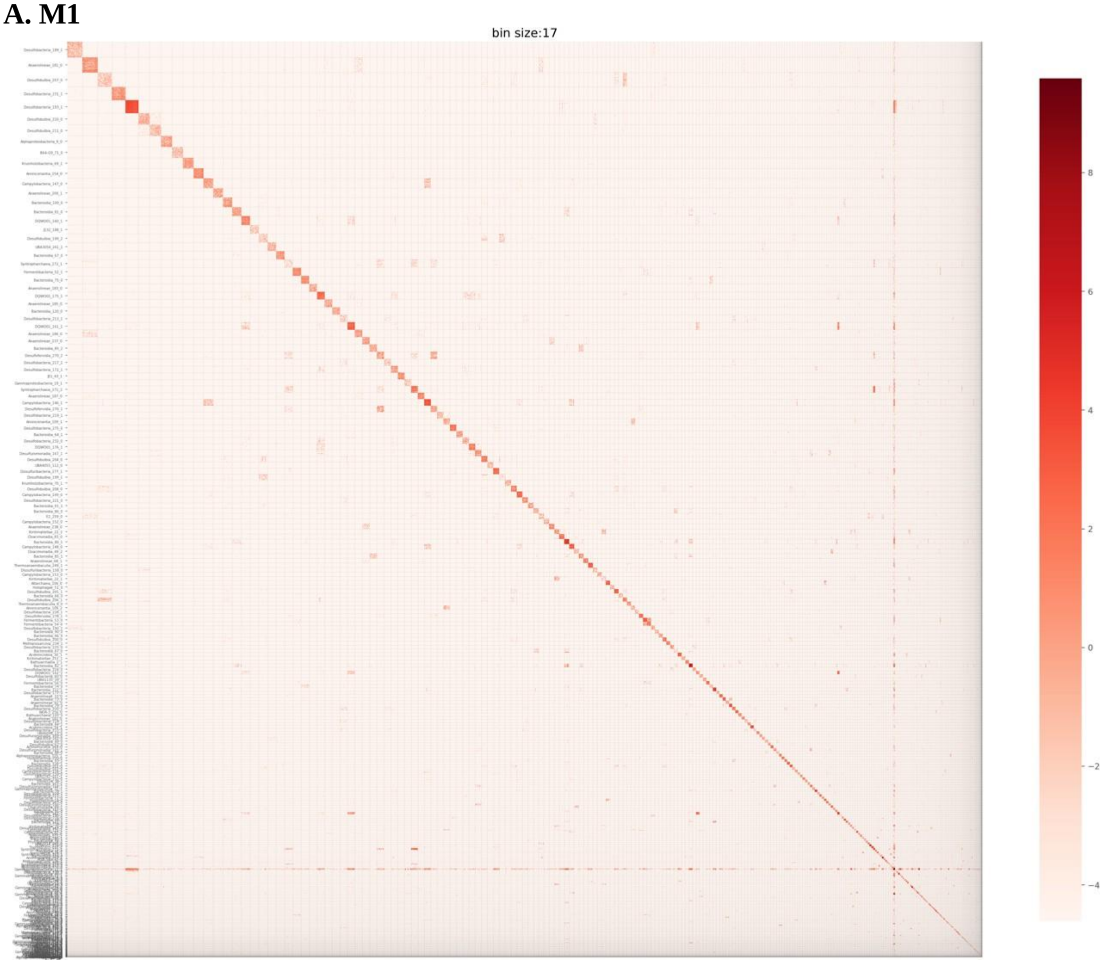

**B. M2**

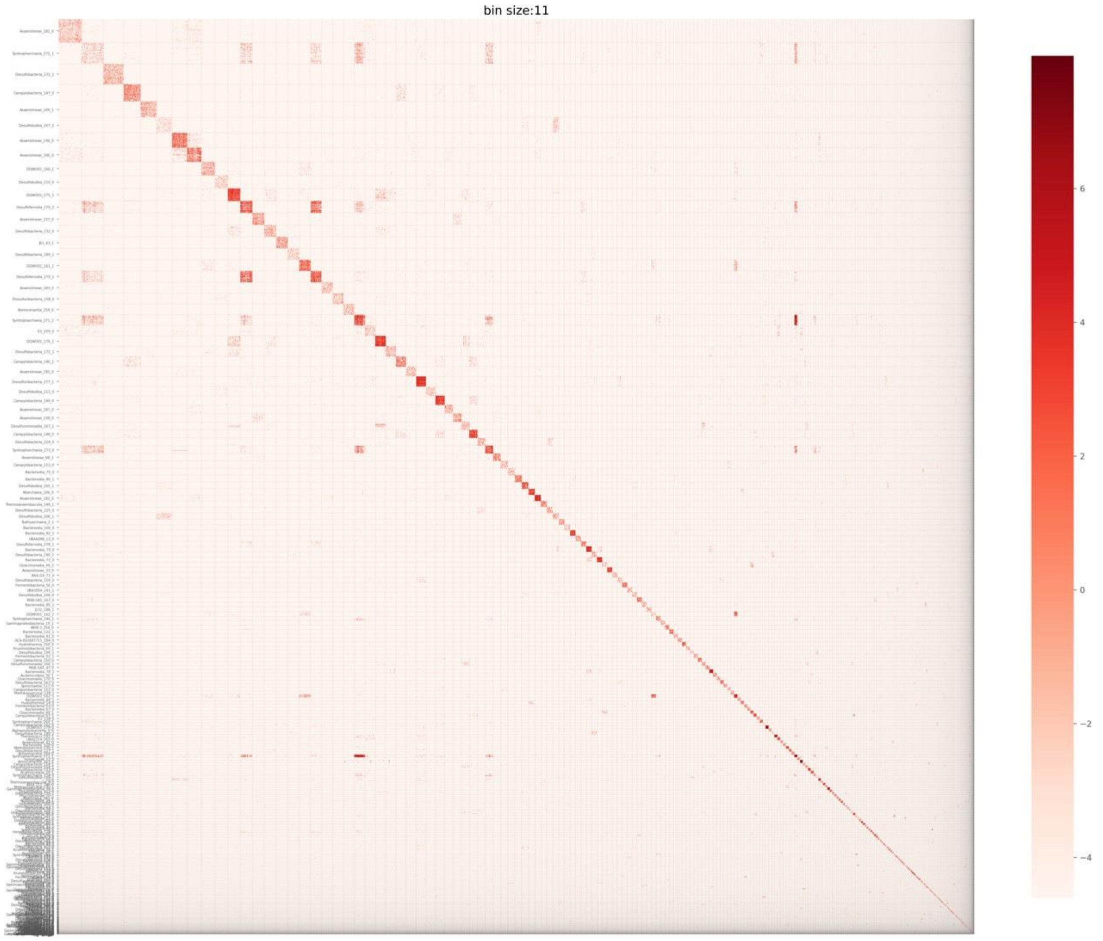

**C. M3**

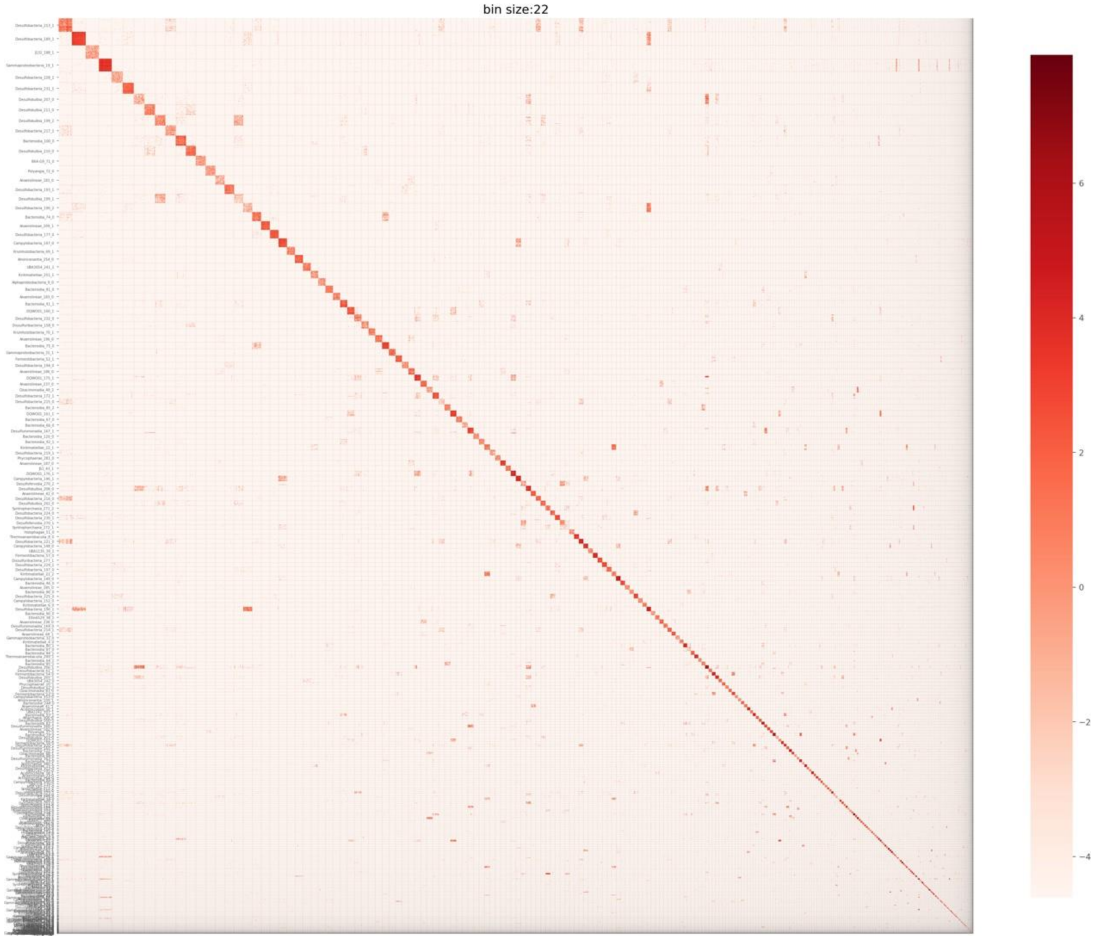

**D. M4**

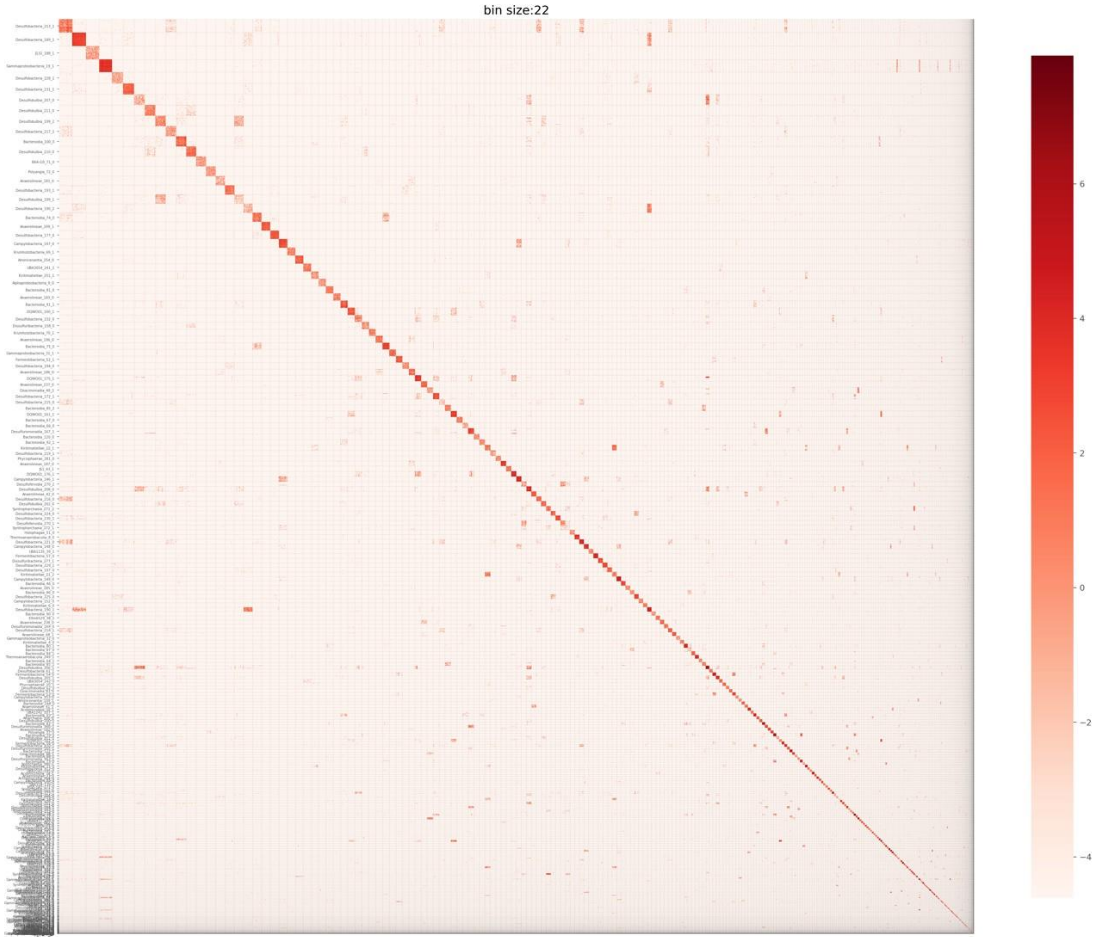

**E. M5**

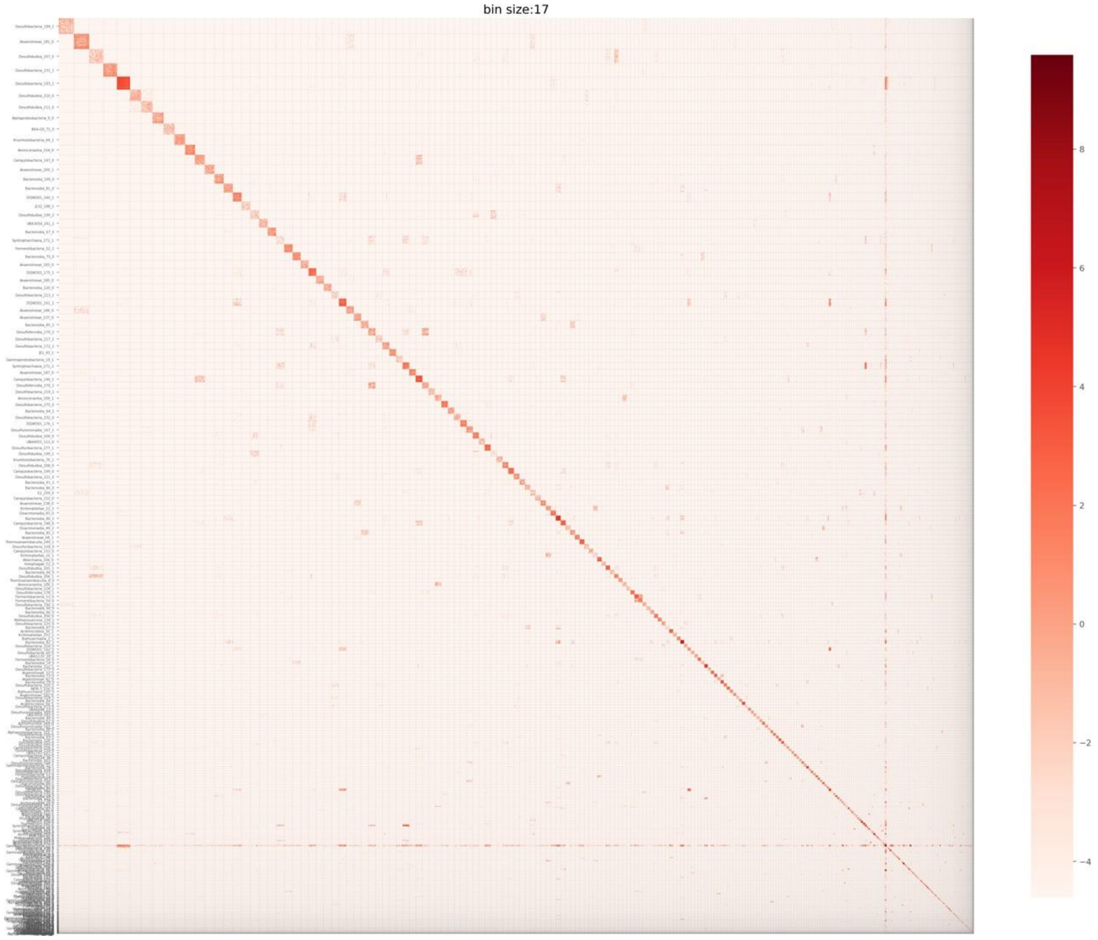

**F. M6**

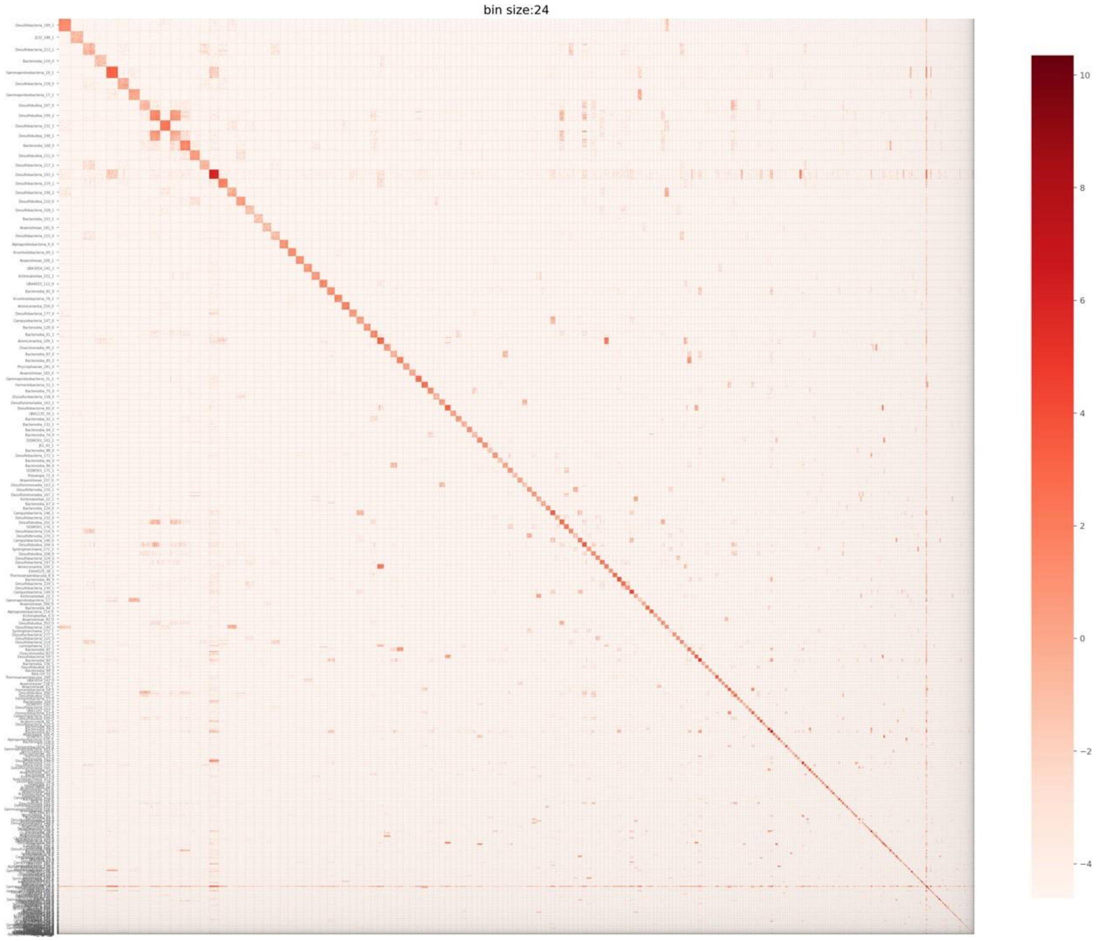

## G. M7

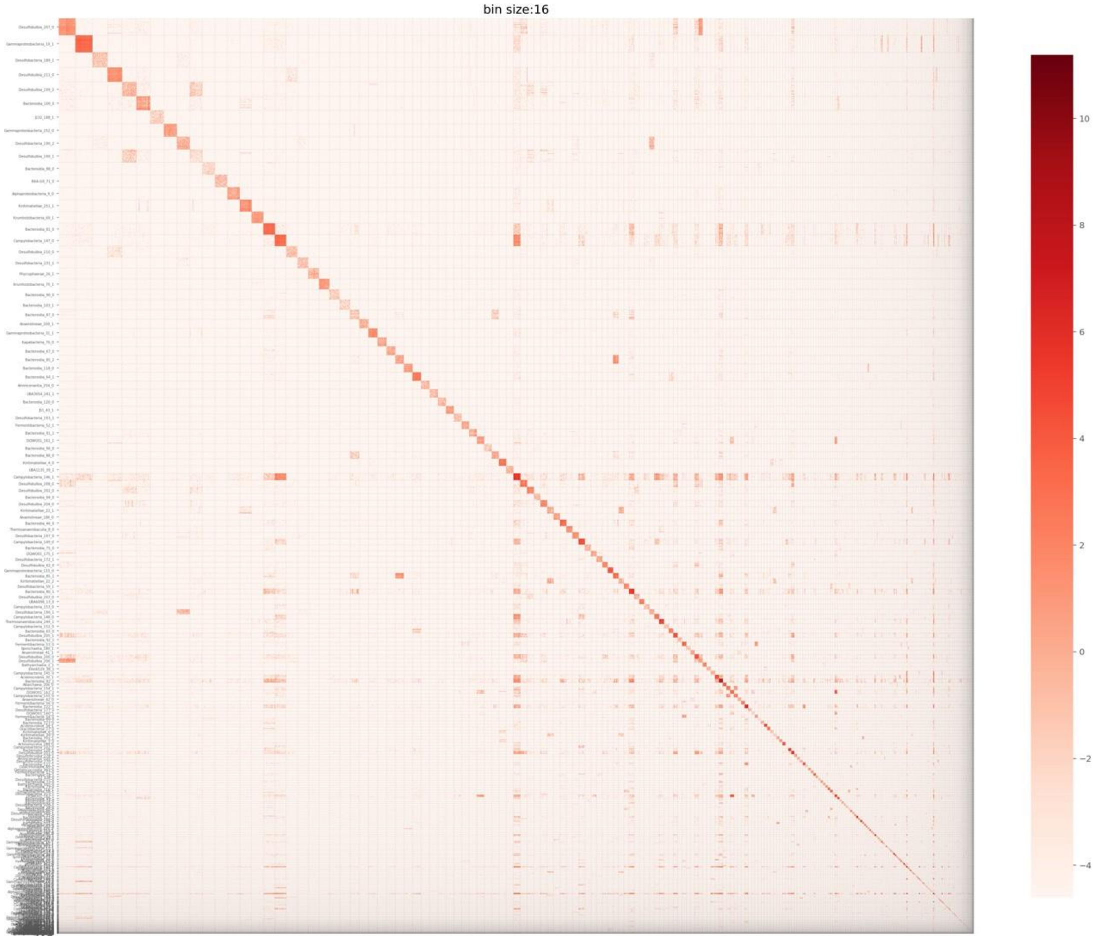

**H. M8**

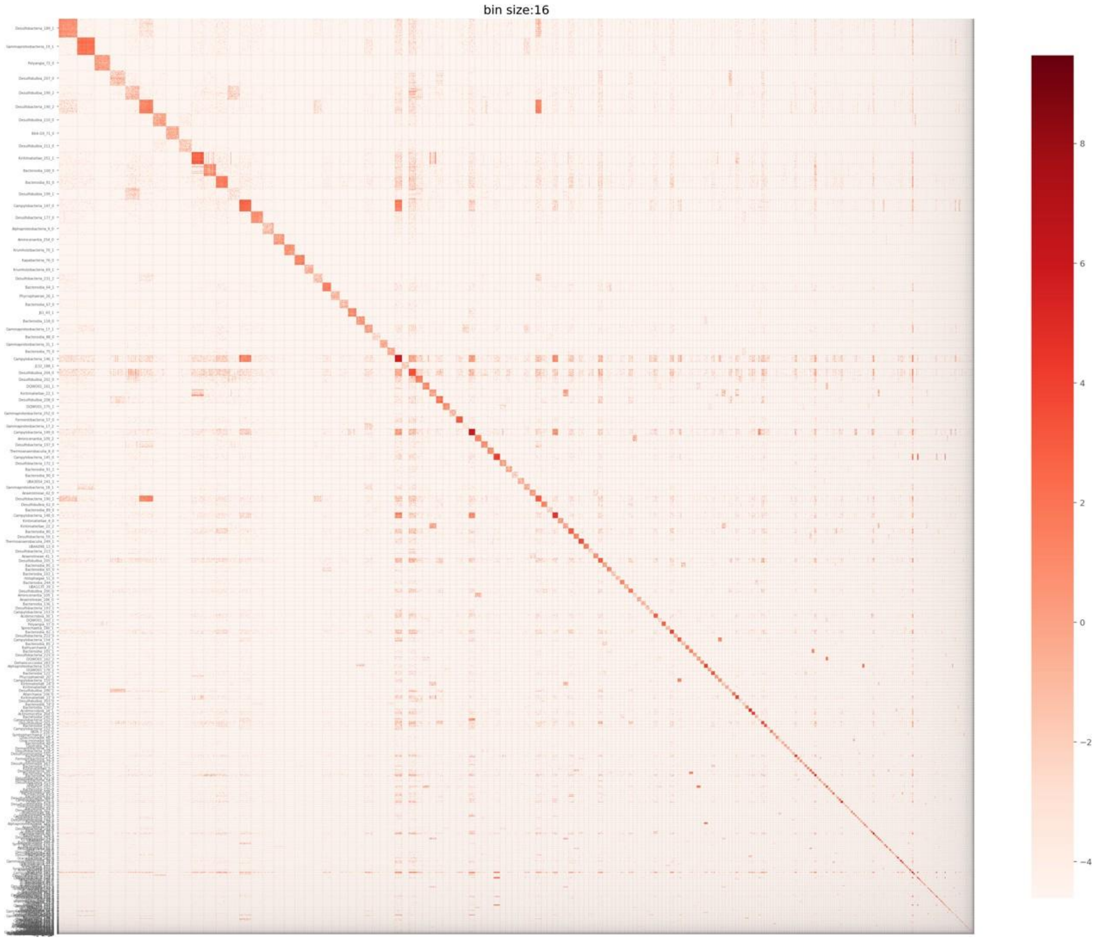

**I. M9**

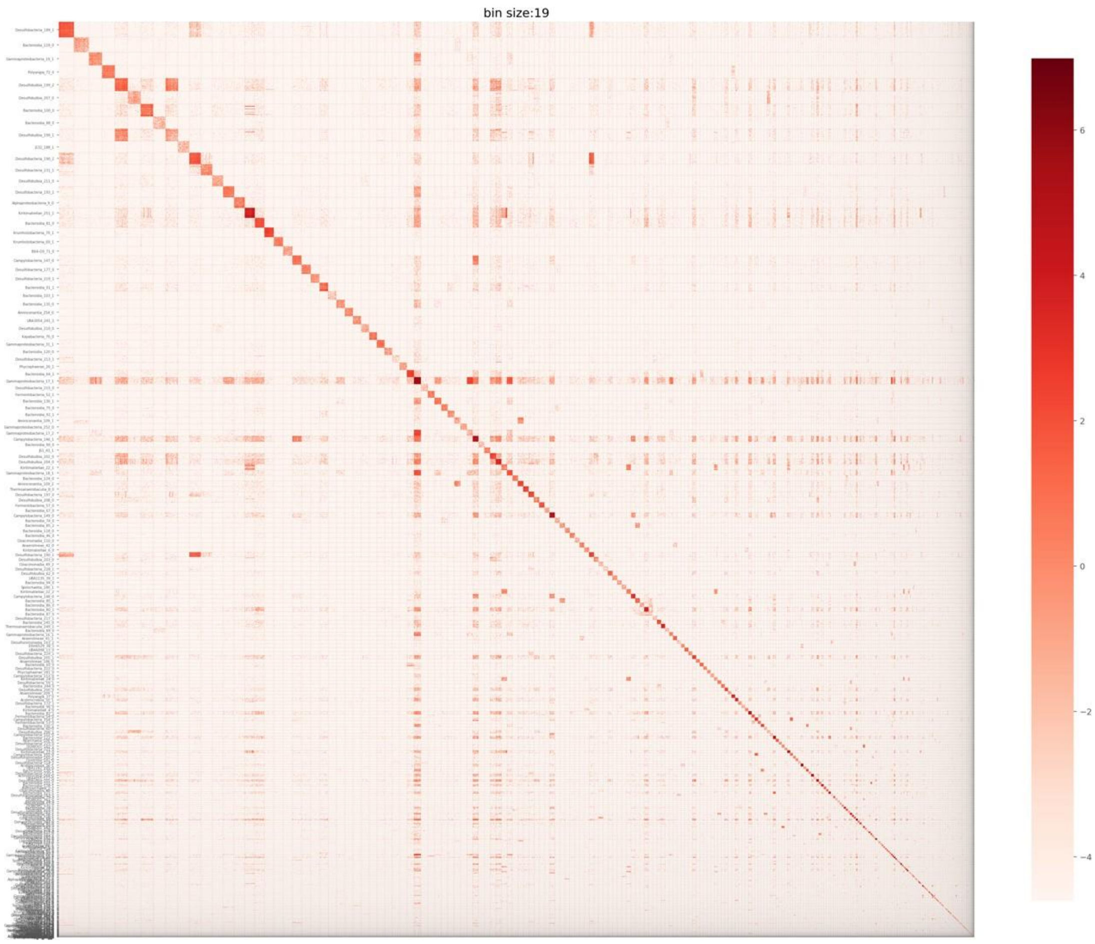

**J. M10**

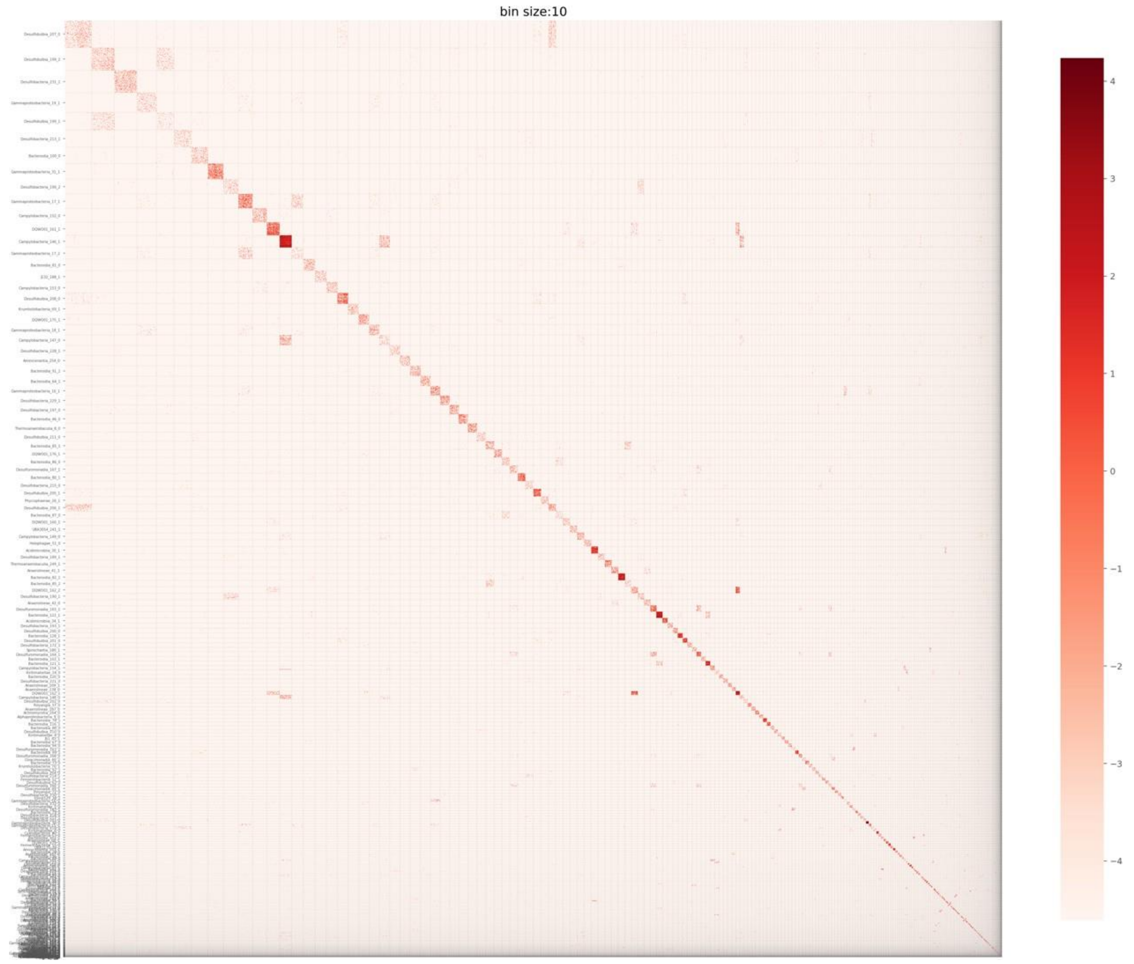


**Figure S1A-J. Hi-C mapping based contact matrices for samples M1-M10.** Normalized contacts were log transformed with $\chi = 0.01$ and matrices were down-sampled using the mean across the bin size. Y-axis labels correspond to the MAG the contig belongs to.
