# Supplementary material for: Viruses interact with hosts that span distantly related microbial domains in dense hydrothermal mats
Source: Nat Microbiol. 2023 Apr 6;8(5):946–57. doi: 10.1038/s41564-023-01347-5 (PMC10159854; doi:10.1038/s41564-023-01347-5)
Supplement: Supplementary file 2 — Reporting Summary [file 41564_2023_1347_MOESM2_ESM.pdf]

nature portfolio

Corresponding author(s): Peter Girguis, Yunha Hwang
Last updated by author(s): Feb 20, 2023

Reporting Summary

Nature Portfolio wishes to improve the reproducibility of the work that we publish. This form provides structure for consistency and transparency in reporting. For further information on Nature Portfolio policies, see our Editorial Policies and the Editorial Policy Checklist.

Statistics

For all statistical analyses, confirm that the following items are present in the figure legend, table legend, main text, or Methods section.

n/a	Confirmed	
☐	☒	The exact sample size (*n*) for each experimental group/condition, given as a discrete number and unit of measurement
☐	☒	A statement on whether measurements were taken from distinct samples or whether the same sample was measured repeatedly
☐	☒	The statistical test(s) used AND whether they are one- or two-sided<br>*Only common tests should be described solely by name; describe more complex techniques in the Methods section.*
☐	☒	A description of all covariates tested
☐	☒	A description of any assumptions or corrections, such as tests of normality and adjustment for multiple comparisons
☐	☒	A full description of the statistical parameters including central tendency (e.g. means) or other basic estimates (e.g. regression coefficient) AND variation (e.g. standard deviation) or associated estimates of uncertainty (e.g. confidence intervals)
☐	☒	For null hypothesis testing, the test statistic (e.g. *F*, *t*, *r*) with confidence intervals, effect sizes, degrees of freedom and *P* value noted<br>*Give P values as exact values whenever suitable.*
☒	☐	For Bayesian analysis, information on the choice of priors and Markov chain Monte Carlo settings
☒	☐	For hierarchical and complex designs, identification of the appropriate level for tests and full reporting of outcomes
☐	☒	Estimates of effect sizes (e.g. Cohen's *d*, Pearson's *r*), indicating how they were calculated

Our web collection on statistics for biologists contains articles on many of the points above.

Software and code

Policy information about availability of computer code

Data collection	No software was used to data collection.
Data analysis	BBduk v37.62 (https://sourceforge.net/projects/bbmap/), sickle v1.33 (https://github.com/najoshi/sickle), metaSPAdes v3.15, CONCOCT v1.1.0, maxBin2 v2.2.7, metabat2 v2.15, ABAWACA v1 (https://github.com/CK7/abawaca), DAS Tool v1.1.2, CheckM v1.1.3, dRep v3.0.1, GTDB-Tk v1.7.0, Prodigal v2.6.3, Diamond v2.0.7, METABOLIC v4, DefenseFinder v1, VirSorter2, VIBRANT v1.2.1, vRhyme v1.1.0, CD-HIT v4.8.1, Bowtie2 v2.3.2, HMMER v3.3.2, MMseqs2 v13.5, CRISPRCasFinder v4.2.20, metaCRAST v1 (https://github.com/molleraj/MetaCRAST), BLAST v.2.6.0, Cytoscape v3.9.1, BWA mem v0.7.17, Phyre2 (http://www.sbg.bio.ic.ac.uk/phyre2), MUSCLE v3.8.31, PyMOL v2.5.1, IQ-Tree v2.0.3, iTOL (itol.embl.de), R v4.0.2, HiCzin v1(https://github.com/dyxstat/HiCzin), inStrain v1.3.1, checkV v0.9.0, vConTACT v0.9.22, ggplot2 v3.3.6, prokka v1.14.6

For manuscripts utilizing custom algorithms or software that are central to the research but not yet described in published literature, software must be made available to editors and reviewers. We strongly encourage code deposition in a community repository (e.g. GitHub). See the Nature Portfolio guidelines for submitting code & software for further information.

Data

Policy information about availability of data

All manuscripts must include a data availability statement. This statement should provide the following information, where applicable:

- Accession codes, unique identifiers, or web links for publicly available datasets
- A description of any restrictions on data availability
- For clinical datasets or third party data, please ensure that the statement adheres to our policy

Sequence data (including raw sequences, assemblies, rep_mMAG and rep_vMAGs) investigated in this study were deposited to NCBI under BioProjects PRJNA879229 [mat samples] and PRJNA879230 [HW samples]. SRA accession numbers are available in Table S1C (shot-gun libraries), Table S9 (Hi-C libraries) and BioSample IDs are listed for rep_mMAGs in Tables S2 and S11, and for rep_vMAGs in Tables S4 and S16. UniRef100 database is accessible at https://www.uniprot.org/help/downloads. IMG/VR database is accessible at https://img.jgi.doe.gov/vr and GOLD database is accessible at https://gold.jgi.doe.gov/. PHROGs (https://phrogs.lmge.uca.fr/) COG-20 (https://www.ncbi.nlm.nih.gov/research/cog-project/), VOG (https://vogdb.org/) databases are available online.

Human research participants

Policy information about studies involving human research participants and Sex and Gender in Research.

Reporting on sex and gender	N/A
Population characteristics	N/A
Recruitment	N/A
Ethics oversight	N/A

Note that full information on the approval of the study protocol must also be provided in the manuscript.

Field-specific reporting

Please select the one below that is the best fit for your research. If you are not sure, read the appropriate sections before making your selection.

☐ Life sciences ☐ Behavioural & social sciences ☒ Ecological, evolutionary & environmental sciences

For a reference copy of the document with all sections, see nature.com/documents/nr-reporting-summary-flat.pdf

Ecological, evolutionary & environmental sciences study design

All studies must disclose on these points even when the disclosure is negative.

Study description	Deep sea hydrothermal mat and water samples were collected for studying the effect of microbial density and syntrophy on microbe-virus interactions in natural microbial communities.
Research sample	Ten deep sea hydrothermal mat samples and ten hydrothermally influenced water samples were collected and the bulk genomic DNA was extracted for sequencing microbial and viral DNA. The mat samples were selected to represent an environment featuring high metabolic interdependence (i.e. syntrophy) and microbial density. The water samples from the physically adjacent hydrothermal plume were used as a comparative sample set featuring microbial community driven by similar metabolisms with lower microbial density.
Sampling strategy	Mat samples were collected using a remotely operated vehicle (ROV Jason) aboard R/V Roger Revelle. We chose the sample size of ten because samples each distanced ~ 70 cm apart sufficiently captured the meso-scale spatial heterogeneity while providing ample biological replication (n=10) in a single contiguous mat. We chose the sample size of ten for hydrothermal water samples in order to conduct statistical comparisons with the mat samples.
Data collection	Mat samples were collected using a remotely operated vehicle (ROV Jason) aboard R/V Roger Revelle, operated by ROV pilots aboard with sampling direction by Yunha Hwang and Peter Girguis. Yunha Hwang recorded the sample log.
Timing and spatial scale	All mat samples were samples were taken during a single dive (ID : J2-1398) on 28 November 2021. Mat samples were collected equidistantly along a transect across a single contiguous mat providing biological replicates of a single mat, while capturing the heterogeneity within the mat. All water samples were taken over two CTD dives over two days 17-18 November 2021 and two ROV dives on 19 November 2021 and 28 November 2021. Water samples were collected in a plume derived from a single source, plumes samples were taken at different distances from the source, featuring different hydrothermal fluid concentrations, to capture the heterogeneity and variation in hydrothermally influenced water microbial communities while keeping the source fluid chemistry constant.

Data exclusions	No data was excluded.
Reproducibility	All computational analyses were conducted using open source softwares with versions and any flags specified and can be reproduced accordingly.
Randomization	Randomization is not relevant for this study because the aim of the study is to characterize and compare the host-virus interactions in microbial communities of high and low microbial density environments and the sequences represent the random sample of the microbial community.
Blinding	Blinding is not relevant for this study because the aim of the study is to characterize and compare the host-virus interaction in microbial communities of high and low microbial density environments and the researchers were blind to the microbial community composition during samples collection and sequencing.

Did the study involve field work? ☒ Yes ☐ No

Field work, collection and transport

Field conditions	Field work was conducted aboard R/V Roger Revelle in the southern Guaymas Basin. Samples were collected on clear days with no documented precipitation. In situ sediment and water temperatures for each sample can be found in Tables S1 and S10.
Location	Microbial mat samples were collected during a research expedition RR2107 on R/V Roger Revelle to the southern Guaymas Basin using remotely operated vehicle Jason on dive J2-1398 on 28 November 2021. Ten pushcore samples were taken across a ~10m wide microbial mat at coordinates 27.00647191°N, 111.40935798°W, at water depth of 2005.3 m. Eight plume water (PW1-PW8) samples were collected during the same research expedition as the mat samples near a pre-identified hydrothermal vent source (27.40921631°N, 111.38910334°W, water depth 1810 m) using a CTD-rosette system (Sea-Bird, Bellevue, WA, USA) at water depths between 1302 m and 1866 m on 17-18 November 2021. PW10 and MOW samples were taken using the 5 L-capacity Niskin bottle on the ROV Jason near the source of the hydrothermal activity (at water depth 1792 m, on 19th Nov 2021) and above the sampled hydrothermal mat (at water depth 2005 m on 28th Nov 2021) respectively.
Access & import/export	Marine science research (MSR) permit (Autorizacion EG0072021) was issued by the Mexican National Institute of Statistics and Geography (INEGI) on 21 July 2021 for the sample collection and scientific activities in the fieldwork location.
Disturbance	Minimal damage was conducted when sampling through the usage of pushcores and the rosette water sampler.

Reporting for specific materials, systems and methods

We require information from authors about some types of materials, experimental systems and methods used in many studies. Here, indicate whether each material, system or method listed is relevant to your study. If you are not sure if a list item applies to your research, read the appropriate section before selecting a response.

Materials & experimental systems

n/a	Involved in the study	
☒	☐	Antibodies
☒	☐	Eukaryotic cell lines
☒	☐	Palaeontology and archaeology
☒	☐	Animals and other organisms
☒	☐	Clinical data
☒	☐	Dual use research of concern

Methods

n/a	Involved in the study	
☒	☐	ChIP-seq
☒	☐	Flow cytometry
☒	☐	MRI-based neuroimaging
